# Pandemic Paradox: Early Life H2N2 Pandemic Influenza Infection Enhanced Susceptibility to Death during the 2009 H1N1 Pandemic

**DOI:** 10.1128/mBio.02091-17

**Published:** 2018-01-16

**Authors:** Alain Gagnon, Enrique Acosta, Stacey Hallman, Robert Bourbeau, Lisa Y. Dillon, Nadine Ouellette, David J. D. Earn, D. Ann Herring, Kris Inwood, Joaquin Madrenas, Matthew S. Miller

**Affiliations:** a Department of Demography, Université de Montréal, Montréal, Canada; b Université de Montréal Public Health Research Institute (IRSPUM), Montréal, Canada; c Statistics Canada, Ottawa, Canada; d Department of Mathematics and Statistics, McMaster University, Hamilton, Canada; e Michael G. DeGroote Institute for Infectious Diseases Research, McMaster University, Hamilton, Canada; f Department of Anthropology, McMaster University, Hamilton, Canada; g Department of Economics and Finance, Department of History, University of Guelph, Guelph, Canada; h Los Angeles Biomedical Research Institute at Harbor, UCLA Medical Center, Los Angeles, California, USA; i Department of Biochemistry and Biomedical Sciences, McMaster Immunology Research Centre, McMaster University, Hamilton, Canada; Mailman School of Public Health, Columbia University

**Keywords:** influenza virus, mortality, pandemics, susceptibility

## Abstract

Recent outbreaks of H5, H7, and H9 influenza A viruses in humans have served as a vivid reminder of the potentially devastating effects that a novel pandemic could exert on the modern world. Those who have survived infections with influenza viruses in the past have been protected from subsequent antigenically similar pandemics through adaptive immunity. For example, during the 2009 H1N1 “swine flu” pandemic, those exposed to H1N1 viruses that circulated between 1918 and the 1940s were at a decreased risk for mortality as a result of their previous immunity. It is also generally thought that past exposures to antigenically dissimilar strains of influenza virus may also be beneficial due to cross-reactive cellular immunity. However, cohorts born during prior heterosubtypic pandemics have previously experienced elevated risk of death relative to surrounding cohorts of the same population. Indeed, individuals born during the 1890 H3Nx pandemic experienced the highest levels of excess mortality during the 1918 “Spanish flu.” Applying Serfling models to monthly mortality and influenza circulation data between October 1997 and July 2014 in the United States and Mexico, we show corresponding peaks in excess mortality during the 2009 H1N1 “swine flu” pandemic and during the resurgent 2013–2014 H1N1 outbreak for those born at the time of the 1957 H2N2 “Asian flu” pandemic. We suggest that the phenomenon observed in 1918 is not unique and points to exposure to pandemic influenza early in life as a risk factor for mortality during subsequent heterosubtypic pandemics.

## IMPORTANCE

The relatively low mortality experienced by older individuals during the 2009 H1N1 influenza virus pandemic has been well documented. However, reported situations in which previous influenza virus exposures have enhanced susceptibility are rare and poorly understood. One such instance occurred in 1918—when those born during the heterosubtypic 1890 H3Nx influenza virus pandemic experienced the highest levels of excess mortality. Here, we demonstrate that this phenomenon was not unique to the 1918 H1N1 pandemic but that it also occurred during the contemporary 2009 H1N1 pandemic and 2013–2014 H1N1-dominated season for those born during the heterosubtypic 1957 H2N2 “Asian flu” pandemic. These data highlight the heretofore underappreciated phenomenon that, in certain instances, prior exposure to pandemic influenza virus strains can enhance susceptibility during subsequent pandemics. These results have important implications for pandemic risk assessment and should inform laboratory studies aimed at uncovering the mechanism responsible for this effect.

## INTRODUCTION

Influenza A virus (IAV) continues to pose one of the most pressing threats to global public health due to its propensity to cause pandemics (1, 2). Over the course of the past 100 years alone, at least five such pandemics have occurred, including the 1918 H1N1 “Spanish flu,” the 1957 H2N2 “Asian flu,” the 1968 H3N2 “Hong Kong flu,” the 1977 “Russian flu,” and the 2009 H1N1 “swine flu” (3). Unlike seasonal influenza virus epidemics, which occur as a result of point mutations in the viral hemagglutinin (HA) protein that permits escape from preexisting antibodies (a phenomenon called antigenic drift), pandemics occur when a novel virus emerges from a reassortment between two or more strains of influenza viruses. Usually, humans have little to no preexisting immunity to these viruses, which originate from different species (“antigenic shift”). For instance, the H1N1 virus responsible for the 2009 “swine flu” pandemic carried a mix of swine, avian, and human influenza virus segments (4). As a result, pandemic viruses often cause more severe illness and deaths than their seasonal counterparts (5), especially in the younger portions of the population, who are unlikely to have been exposed to ancestral strains.

Preexposure to antigenically related IAV strains in older cohorts has had a protective effect on those cohorts during subsequent pandemics. During the 1918 H1N1 “Spanish flu” pandemic, those born prior to 1890 are thought to have been partially protected by a putative H1 virus that circulated prior to the H3Nx pandemic that occurred that year (6–9). Similar protection has also been reported during the 1968 H3N2 “Hong Kong flu” pandemic for those exposed to the 1890 “Russian flu” pandemic, which is thought to have been caused by an H3Nx-like virus (7, 10), and again during the 2009 H1N1 “swine flu” pandemic for those exposed to 1918 H1N1 or immunized against the 1976 “Fort Dix, New Jersey” H1N1 virus (11–13). Exposure to heterosubtypic viruses (within the same HA group) has also been shown in boost titers of broadly neutralizing antibodies (bnAbs) that bind to the HA stalk domain (14–16), which at least partly account for the levels of protection observed among the elderly in many instances.

Titers of bnAbs are known to be higher in the elderly as a result of repeated exposures to the mosaic of strains that circulated in the past. Yet, as was demonstrated recently for Mexico (17, 18), protection among the elderly usually vanishes quickly a few years after a pandemic outbreak. Our current study shows a similar decrease in protection among the elderly during the 2013–2014 H1N1 outbreak that followed the 2009 pandemic in the United States and Mexico. However, it also reveals an increased susceptibility to death, both in 2009 and in 2013–2014 influenza season, for middle-aged groups that cannot be interpreted as a mere “age shift” in the levels of protection against the circulating virus but rather as an increased susceptibility to the new strain for specific cohorts exposed early in life to a highly dissimilar pandemic strain.

While most IAV preexposures are thought to have either a net positive or neutral impact on future infections, there are at least two earlier examples for which several groups have suggested that prior exposure to IAV may have been deleterious. During the 1918 Spanish flu pandemic, the peak mortality occurred for those around 28 years of age (6–9). For individuals who were born in 1890, the first IAV to which these individuals would have been exposed was the putative H3Nx 1890 “Russian flu” pandemic virus (19). A peak in mortality was also observed during the 1968 H3N2 pandemic for those born at the time of the 1918 H1N1 Spanish flu (7). Several hypotheses have been advanced to explain this phenomenon, including T cell-mediated immunopathology (6, 20), antigenic imprinting (6, 8, 21), and developmental abnormalities as a result of exposure to influenza virus while *in utero* (6, 8) or as a neonate (6, 8, 22). However, most of these hypotheses remain untested.

To determine whether other similar age-related effects may have occurred, we calculated influenza mortality by year of birth in the United States and Mexico during the 2009 H1N1 pandemic and during the subsequent H1N1 outbreak of 2013 to 2014. Accordingly, we observed peaks in death counts, rates, and ratios from influenza for those who were 52 years of age during the 2009 H1N1 IAV pandemic in both countries. This corresponds to a birth year of 1957, which was the year of the H2N2 “Asian flu” pandemic. Our data provide a new example of a scenario wherein those born during an influenza pandemic experienced elevated mortality during a subsequent, heterosubtypic influenza pandemic.

## RESULTS

**FIG 1:**
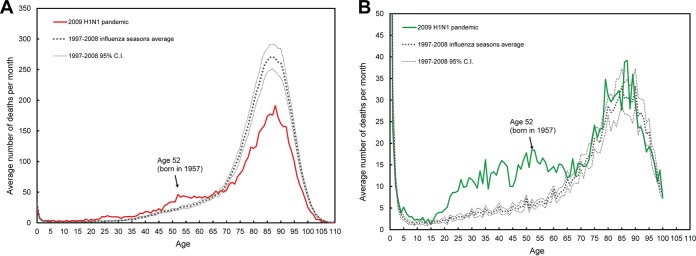
Average number of deaths from pneumonia and influenza (P&I) during the 2009 H1N1 influenza pandemic and during seasonal outbreaks from 1997 to 2008 in the United States (A) and in Mexico (B). Age is shown in years on the *x* axes. 95% C.I., 95% confidence interval.

To assess the specific age-related mortality caused by the 2009 H1N1 “swine flu” pandemic, we first determined pneumonia and influenza (P&I) average mortality counts per month by single-year age during the outbreak from September 2009 to January 2010 and for all influenza seasons (November to April) from the 1997–1998 influenza season to the 2007–2008 influenza season in the United States and Mexico (Fig. 1). The standard deviations used to build the confidence intervals around the seasonal averages were based on the rate of P&I deaths of the 11 seasons. The sensitivity analyses in the supplemental material present confidence intervals for seasonal values as well as for the H1N1 outbreaks using P-splines, thus providing a means to assess the precision of these results (see Fig. S1 in the supplemental material). This section of the supplemental material also presents SiZer plots, which help provide statistical support in the identification of regions of increase or decrease, as well as local maxima in the data (Fig. S3).

10.1128/mBio.02091-17.2 FIG S1  Average number of deaths per month from pneumonia and influenza (P&I) during the 2009 influenza pandemic, the 2013–2014 resurgent H1N1 influenza epidemic, and during the 1997–2008 influenza seasons in the United States (A and B) and Mexico (C and D). Smoothed lines and 95% confidence intervals were obtained by P-spline smoothing based on penalized Poisson likelihood. Download FIG S1, TIF file, 9.61 MB. Copyright © 2018 Gagnon et al. 2018 Gagnon et al. This content is distributed under the terms of the Creative Commons Attribution 4.0 International license.

As has been reported earlier (23), we observed lower mortality from P&I for the elderly during the 2009–2010 pandemic compared to previous influenza seasons in the United States. However, we also observed a surprising increase in mortality counts for the individuals who were approximately 45 to 60 years old (born between 1949 and 1964). Closer inspection shows that a peak in mortality counts for this cohort occurred for those who were 52 years of age, that is, for those born in 1957, the year of the H2N2 Asian influenza pandemic (Fig. 1A). The elevation in mortality for those up to 60 years old suggests that early life exposure to the H2N2 pandemic strain was sufficient to elevate risk, even if it was not necessarily their first exposure to influenza virus. This peak is apparent, though less obvious in Mexico (Fig. 1B), for which there are also noticeable increases in the number of P&I deaths among younger individuals, especially infants, but also among people born at the time of the 1968 pandemic.

**FIG 2:**
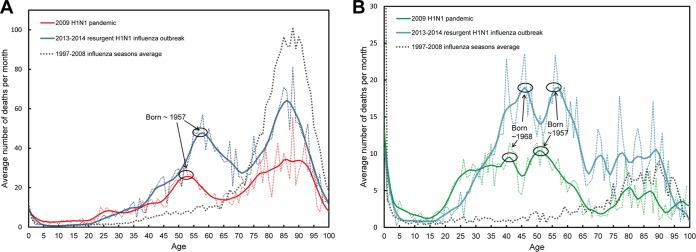
Average number of influenza deaths per month estimated from Serfling models in the United States (A) and in Mexico (B). (A) For the U.S. data, the months when mortality was higher than the “epidemic thresholds” established by the CDC were used to define epidemic periods (see Materials and Methods), i.e., from October to January 2010 for the 2009 pandemic and from December 2013 to March 2014 for the 2013–2014 H1N1 outbreak. (B) For the Mexican data, the months chosen to define epidemic periods were those with noticeable increases in the number of severe acute respiratory infections (SARI) cases in reference 18.

Next, we analyzed excess mortality (count) estimates for each age using the Serfling method (see Materials and Methods) for the 2009 pandemic, adding the resurgent 2013–2014 influenza season estimates (24) (Fig. 2). Due to the fluctuation of the curves at certain ages, we smoothed the data. For simplicity and to ease visualization, we first used locally weighted scatterplot smoothing (lowess) to plot the smoothed curves. Alternative methods of smoothing are presented in the supplemental material, including confidence intervals, which confirm the trends presented here (see Fig. S2 and S4).

10.1128/mBio.02091-17.3 FIG S2  Average number of deaths per months estimated from the surveillance-Serfling (A and B) and Serfling (C and D) models for the United States (A to D) and Mexico (E and F). Poisson P-splines were used to smooth death count estimates. Shaded areas depict 95% confidence intervals, which account for variation in both Serfling and P-splines models. Download FIG S2, TIF file, 14.0 MB. Copyright © 2018 Gagnon et al. 2018 Gagnon et al. This content is distributed under the terms of the Creative Commons Attribution 4.0 International license.

10.1128/mBio.02091-17.4 FIG S3  Average number of deaths per month from P&I and SiZer plots of the first derivatives of polynomial smoothings of these estimates for the United States (A and B) and Mexico (C and D) in 2009 (A and C) and 2013–2014 influenza season (B and D). The age-specific death counts used to build polynomial smoothings in the top figures are plotted as colored circles, along with the 95% confidence intervals in gray. In the SiZer plots (bottom figures), the color scheme is blue in the locations where the curve is significantly increasing, red when it is significantly decreasing, and purple where it cannot be declared whether it is increasing or decreasing. Areas where the data are too sparse to make a statement about significance (the polynomial weights rely on too few points, i.e., the effective sample size in the window is less than 5) are shown in light gray. The horizontal line at h = log(0.6) = 4 in the SiZer plots represents a bandwidth of 4, used to create the polynomial smoothing in the top figure of each panel. The white curves in the SiZer plots provide a sense of the window widths for each bandwidth; the horizontal distance between the two lines is 2 h (i.e., plus 2 standard deviations of the Gaussian kernel). Download FIG S3, TIF file, 19.0 MB. Copyright © 2018 Gagnon et al. 2018 Gagnon et al. This content is distributed under the terms of the Creative Commons Attribution 4.0 International license.

10.1128/mBio.02091-17.5 FIG S4  Average number of deaths per month estimated from surveillance-Serfling and Serfling models and SiZer plots of the first derivatives of polynomial smoothings of these estimates in United States and Mexico for 2009 and 2013–2014 influenza season. The age-specific death counts used to build polynomial smoothings in the top figures are plotted as colored circles, along with the 95% confidence intervals in gray. In the SiZer plots (bottom figures), the color scheme is blue in the locations where the curve is significantly increasing, red when it is significantly decreasing, and purple where it cannot be declared whether it is increasing or decreasing. Areas where the data are too sparse to make a statement about significance (the polynomial weights rely on too few points, i.e., the effective sample size in the window is less than 5) are shown in light gray. The horizontal line at h = log (0.6) = 4 in the SiZer plots represents a bandwidth of 4, used to create the polynomial smoothing in the top figure of each panel. The white curves in the SiZer plots provide a sense of the window widths for each bandwidth; the horizontal distance between the two lines is 2 h (i.e., plus 2 standard deviations of the Gaussian kernel). Download FIG S4, TIF file, 19.4 MB. Copyright © 2018 Gagnon et al. 2018 Gagnon et al. This content is distributed under the terms of the Creative Commons Attribution 4.0 International license.

10.1128/mBio.02091-17.6 FIG S5  Case fatality rate ratios (CFRRs) of influenza mortality during the 2009 H1N1 influenza pandemic using alternative attack rates. Ratios of case fatality rates are relative to the average of values for influenza seasons from 1998 to 2008. For both the 2009 pandemic and the flu seasons, age-specific case fatality rates were obtained by dividing death rates by the attack rates provided in Fig. 7 for each age. The log (base 10) is used on the *y* axis in order to preserve symmetry about a case fatality rate ratio of 1 (i.e., the line at zero on a log scale). The gray vertical lines mark the 1918, 1957, and 1968 influenza epidemics. Only smoothed values are presented to ease reading of the figure. Download FIG S5, TIF file, 2.24 MB. Copyright © 2018 Gagnon et al. 2018 Gagnon et al. This content is distributed under the terms of the Creative Commons Attribution 4.0 International license.

In comparison with our prior observations based on simple P&I tabulations, the overall monthly death counts were smaller, since these new estimates should exclude pneumonia deaths which were not caused, in principle, by influenza. The peak in excess mortality observed for those who were 52 years old in 2009 (born in 1957) was also, in relative terms, more pronounced in Fig. 2 than in Fig. 1, while the estimated numbers of deaths at older ages are much lower. These findings are consistent with the peak in excess mortality that was recently reported to occur at 50 to 54 years of age in the United States with a traditional Serfling model (25); we argue in the discussion that the peak at the exact age of 52 years, the midpoint of the 50-to-54 5-year age group, results from an early life exposure to the 1957 H2N2 pandemic virus. The slight elevation in mortality observed for those born in 1968 in Mexico noted in the P&I distribution (Fig. 1B) also becomes much more apparent using the analysis shown in Fig. 2B.

Although it has received considerably less attention, the 2013–2014 H1N1 influenza season was nevertheless at least as deadly as the 2009 pandemic, if not more so. Despite the fact that the estimated number of deaths depends on the period chosen to define the epidemic (Fig. S6), the number of deaths among the elderly and among those aged 50 to 65 years old appeared higher in the 2013–2014 influenza season than in 2009. More importantly, relative to the 2009 pandemic, the peak of mortality among these middle-aged people is shifted to the right by about 4 to 5 years; this is particularly striking in the results for Mexico, for which the shift to the right is apparent for people born in 1968 as well as for people born in 1957 (see ellipses in Fig. 2B).

10.1128/mBio.02091-17.7 FIG S6  Case fatality rate ratios (CFRRs) of influenza mortality during the 2009 H1N1 influenza pandemic using alternative pandemic period definitions. Ratios of case fatality rates are relative to the average of values for influenza seasons from 1998 to 2008. For both the 2009 pandemic and the flu seasons, age-specific case fatality rates were obtained by dividing death rates by the attack rates provided in Fig. 7 for each age. The log (base 10) is used on the *y* axis in order to preserve symmetry about a case fatality rate ratio of 1 (i.e., the line at zero on a log scale). Gray vertical lines mark the 1918, 1957, and 1968 influenza epidemics. Only smoothed values are presented to ease reading of the figure. Download FIG S6, TIF file, 2.29 MB. Copyright © 2018 Gagnon et al. 2018 Gagnon et al. This content is distributed under the terms of the Creative Commons Attribution 4.0 International license.

**FIG 3:**
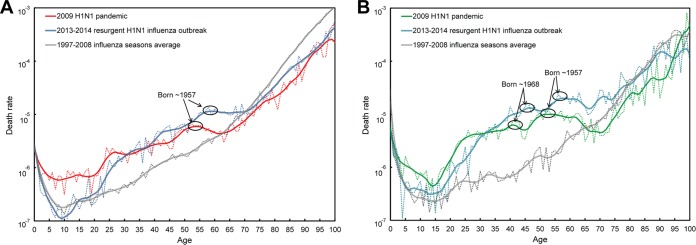
Monthly death rates from influenza as estimated from Serfling models. The death rates were obtained by dividing the estimated number of deaths by the number of individuals in the population at risk during a 1-month period in the United States (A) and Mexico (B).

The surge in mortality in these cohorts was also marked by an ellipse in Fig. 3, which depicts monthly rates of mortality from influenza, calculated by dividing the death counts from Fig. 2 by the population at risk in each 1-year age category (see Materials and Methods). Another local peak of influenza mortality for 24- to 26-year-old individuals was also apparent in Fig. 3. This corresponds to an important antigenic change resulting from the acquisition of a new glycosylation site by H1N1 viruses that circulated after 1985 (26, 27).

**FIG 4:**
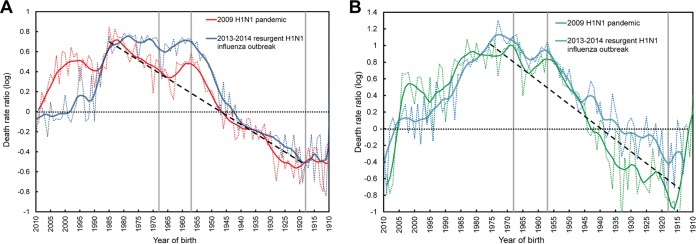
Death rate ratios of influenza mortality during the 2009 H1N1 influenza pandemic and the 2013–2014 seasonal H1N1 outbreaks. Ratios for H1N1 influenza outbreaks in the United States (A) and Mexico (B) are relative to the average of values for influenza seasons from 1997 to 2008. The log (base 10) is used on the *y* axes in order to preserve symmetry about a death rate ratio of 1 (i.e., the line at zero on a log scale). Gray vertical lines mark the 1918, 1957, and 1968 influenza epidemics. The dotted black lines depict the trend of increasing death rate ratios after 1918, as differential protection progressively declines.

Figure 4 illustrates death rate ratios comparing the 2009 and 2013–2014 H1N1 outbreaks to the average seasonal flu from 1997 to 2008. To facilitate interpretation, we depicted birth cohorts on the *x* axis, rather than ages. The similarities between the Mexican and U.S. trends in Fig. 4 are remarkable. As is immediately obvious from Fig. 4, the ratios were quite low for those born at the time of the 1918 pandemic and high at young ages (i.e., for the recent birth cohorts), especially during the 2009 pandemic, a well-known trend (23, 28). Perhaps less well-known was the sudden drop in risk (both in the United States and Mexico) during the 2013–2014 influenza season for cohorts born after 1985. These individuals mounted a protective H1 antibody response focused primarily on an epitope involving residue K133 (26). In contrast, the H1 antibody response of individuals born prior to 1985 was focused on an epitope involving residue K166 of the pandemic H1N1 (pH1N1) virus. When this residue mutated to K166Q in 2013–2014 influenza season, they were once again susceptible to infection (27). A local peak in death rates is again immediately apparent during the 2009 pandemic for those born in 1957, at the time of the H2N2 pandemic. This peak was a deviation from the general decline in protection mediated by preexisting immunity (depicted by hatched black lines, for clarity). Death rates in 2009 then dropped for those born between 1957 and 1968 as H2N2 became epidemic and caused fewer and less severe infections among the population (29). Rates then continued to rise in cohorts born until the mid-1980s when the aforementioned antibody focusing shifted to residue K133 (27).

**FIG 5:**
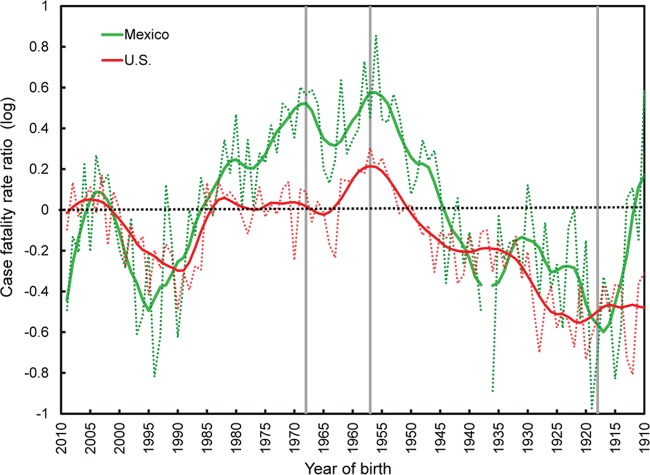
Case fatality rate ratios (CFRRs) of influenza mortality during the 2009 H1N1 influenza pandemic. Ratios of case fatality rates are relative to the average of values for influenza seasons from 1997 to 2008. For both the 2009 pandemic and the reference flu seasons, age-specific case fatality rates were obtained by dividing death rates by attack rates provided in Fig. 7 for each age. The log (base 10) is used on the *y* axis in order to preserve symmetry about a case fatality rate ratio of 1 (i.e., the line at zero on a log scale). Gray vertical lines mark the 1918, 1957, and 1968 influenza epidemics.

Mortality rates from seasonal influenza are usually very low among adolescents and young adults, which primarily accounts for the large risk ratios measured for these ages during the 2009 pandemic. On the other hand, the attack rates for these ages were very high during that pandemic (30), much more so than during usual epidemic seasons (see Fig. 7), and to a point that school closures during the outbreak significantly slowed the spread of the pandemic in Canada (31). In fact, the excess mortality of those who were less than 30 years old was most likely the result of a high attack rate, and not of a high case fatality rate. As seen in Fig. 5, the case fatality rate ratio (CFRR) of cohorts born after about 1980 (i.e., less than 30 years old in 2009) was either lower or approximately equal to 1 in the United States. This CFRR compares the case fatality rate of the 2009 pandemic to that of the previous 10-year average of seasonal outbreaks (see Materials and Methods), meaning that children and young adults in 2009 had an equal or a lower case fatality rate than those who were of the same ages during the previous seasons of influenza. In Mexico, where the 2009 pandemic and the 2013–2014 influenza virus seasons were more severe, the estimated case fatality ratios are larger. Yet, the similarities between the results for two countries are again striking, with the exception of an increased risk in Mexico for the 1968 cohort.

## DISCUSSION

The results of this study are in agreement with previous studies which have reported increased frequencies of cross-reacting antibodies against the H1N1 Cal09 virus in 2009 for individuals born between 1918 and the 1930s (32, 33). This protection, which can be appreciated in Fig. 4, came as a result of exposure to the 1918 H1N1 “Spanish flu” virus, and those viruses that circulated thereafter. Indeed, these viruses shared considerable antigenic similarity with the 2009 H1N1 virus (4).

However, the peak in excess mortality for individuals born in 1957 during the 2009 “swine flu” pandemic cannot be fully explained by differential protection conferred by preexisting immunity (see peaks above the dashed lines in Fig. 4). Although there is a general trend of increasing mortality for those whose birth years left them less likely to have been infected by a 1918-like H1N1 virus at a young age, it is clear that H1N1 disappeared from circulation upon emergence of the H2N2 pandemic strain in 1957 (19). Those born prior to 1957 would have experienced some protection conferred by prior exposure to H1N1 viruses, but the peak in excess mortality declines sharply for those born between 1958 and 1968 despite no further circulation of H1N1 viruses until their reappearance in 1977. This is in close agreement with an interesting study that showed a similar peak in influenza morbidity for those born around 1957, followed by a steep drop for those born between 1958 and 1968 (34). This observation is also consistent with historical data which reported both declining rates and severity of H2N2 infection in the years after 1957 (29). This phenomenon has also been reported for H1N1 viruses and has been ascribed, at least in part, to the accumulation of glycosylation sites (35). Together, these factors would have attenuated the strength of H2N2 “imprinting” at both the individual and population levels in the years following 1957.

The peaks in excess mortality observed during the 2009 H1N1 pandemic and 2013–2014 H1N1 epidemic year for those born in 1957 also mirror the mortality trend observed during the 1918 Spanish flu for those born during the 1890 flu pandemic (6–9). The data reported here thus demonstrate the susceptibility associated with birth during the year of an influenza virus pandemic was not unique to 1890 and 1918 and represent a previously unappreciated risk factor during subsequent pandemics. The recurrence of this trend, which has all of the hallmarks of a cohort effect (9), highlights the value of studying influenza mortality figures using single-year age data, instead of the very large age groups that are in use in most, if not all, flu sentinel programs (e.g., 0 to 4, 5 to 17, 18 to 64, and 65+ years old). In some cases, the 0-to-64 age group is simply compared with individuals who are more than 65 years old, a classification which fails to reveal any eventual cohort effects.

Yet, in contrast to what was observed in Mexico, a mortality peak was not evident in the United States in the 2009 pandemic or the 2013–2014 influenza season for those born in 1968, the year of the H3N2 “Hong Kong flu” pandemic—a scenario that would have been more analogous to that which occurred in 1918 for those born in 1890 (6). This discrepancy between the U.S. and Mexican data could be explained by the fact that the 1968 H3N2 and 2009 H1N1 pandemics were separated by a vast U.S. vaccination campaign against swine flu in 1976. Exposure to the 1976 swine vaccine relatively early in life may have resulted in more robust immunity against H1N1 viruses in general compared to those who were born in 1957, who would have thus had much longer influenza virus exposure history before receiving the vaccine.

Following the 2009–2010 influenza season where pH1N1 caused the vast majority of infections, H3N2 viruses became the predominant source of infections between the 2010–2011 and 2012–2013 seasons. During the 2013–2014 influenza season though, pH1N1 reemerged as the dominant cause of infections. Relative to the parental pH1N1 that emerged in 2009, the H1N1 strain that circulated in the 2013–2014 season was found to carry a K166Q mutation in HA. Many middle-aged adults were found to have antibodies specific for K at position 166, due to their exposure to seasonal H1N1 viruses that circulated between 1977 and 1985 (27). Conversely, those born after 1985 mounted an antibody response focused on residue K133, and indeed experienced reduced mortality in the 2013–2014 influenza season relative to the 2009 influenza season (26). High attack rates in this cohort during the 2009 pandemic (as shown in Fig. 7) may have also resulted in more robust population-level immunity. The replacement of K with Q in the 2013–2014 influenza season is postulated to have reduced the protective immunity of middle-aged adults (who were primed to K166), and may thus explain the elevated death counts that we observed for these cohorts in the 2013–2014 influenza season relative to the 2009 season (36). These trends were remarkably consistent in both the United States and Mexico (Fig. 3 and 4).

Strain-specific antibodies have historically been considered the primary correlate of protection against influenza viruses (37). Elicitation of these antibodies is therefore the goal of current-generation influenza virus vaccines. Unfortunately, the strain-specific nature of these vaccines does not offer protection against the emergence of pandemic influenza virus strains, as was clearly demonstrated during the 2009 H1N1 “swine flu” outbreak (38). This has highlighted the urgent need to develop more broadly protective “universal” influenza virus vaccines—capable of providing protection against both seasonal and pandemic influenza strains (2, 39). Recently, Gostic et al. (40) argued that imprinting conferred by the first influenza virus to which an individual is exposed provides lifelong cross-protective immunity to highly pathogenic avian influenza strains from the same HA group. This was demonstrated using aggregate data from H5N1 and H7N9 infections in the isolated regions where these epidemics have occurred. While this phenomenon seems to hold true in many scenarios, our data suggest that this principle is not universal.

The model set forth by Gostic et al. (40) would predict that during historical pandemics, individuals first exposed to an influenza virus from within the same HA group as the pandemic strain should be “protected” relative to individuals first exposed to a strain belonging to a different HA group. Yet, as was observed in this study, people born at the time of the 1957 H2N2 influenza pandemic (thus primed to a group 1 HA) were at an increased risk of death during the 2009 H1N1 pandemic and the 2013–2014 resurgent H1N1 outbreak, which were also caused by a group 1 HA virus. Indeed, the CFRR during the 2009 H1N1 pandemic of those born during the circulation of H2N2 (a group 1 HA virus) noticeably exceeded that of those born between 1968 and 1977, most of whom would have been likely primed by H3N2 (a group 2 virus). Of course, the 2009 H1N1 pandemic was somewhat unique, in that almost the entire population had some degree of pre-exposure to H1N1 viruses that circulated previously—resulting in varying degrees of cross-protection based on the antigenic similarity between the H1N1 strain(s) to which cohorts were exposed and the pandemic strain itself.

When observed closely, data from Gostic et al. (40) show evidence that early life exposure to the 2009 pH1N1 may have led to higher increases in the numbers of cases and deaths during the following years from H5N1 than from H7N9, even though the former is from group 1, while the latter is from group 2. We would suggest that the increases in risk of severe infection from both H5N1 and H7N9 for the children born at the time of the 2009 pandemic are the result of their commitment early in life to a heterosubtypic pandemic influenza virus (i.e., H1N1) relative to both these two viruses.

It could also be suggested that the mortality peak observed for the cohorts born between 1945 and 1965 is due to the higher susceptibility (by some unknown mechanism) of the baby boomers. Incidentally, the 1957 cohort is one of the largest cohorts of the 20th century in the United States, and Lexis surfaces of all-cause mortality indicate an increased mortality for cohorts centered around that year, beginning in the period from 1980 to 1985 and persisting into the new millennium (41). In general, large cohorts have fewer per capita resources, which could lead to increased levels of frailty that pandemic outbreaks or severe influenza seasons would reveal with acuity, typical of a harvesting effect. However, breaking down the analyses by specific causes of death and using Serfling models applied to pneumonia and influenza, no large increase of mortality from influenza itself is seen for these cohorts during all of those years (42), some of which were appreciably more deadly than the 2009 and 2013–2014 outbreaks. In other words, the excess influenza mortality centered on the 1957 generation is observed for the 2009 pandemic and, perhaps less clearly, during the 2013–2014 outbreak, but not during previous, more severe influenza seasons. In addition, during the 1968 H3N2 pandemic, children aged 11 years old and thus born during the 1957 H2N2 pandemic did not experience notable excess mortality (7). As indicated above, death rate ratios from pneumonia and influenza mortality in 1968 peaked instead for the cohorts born at the time of the 1918 pandemic, which were primed early in life to a doubly heterosubtypic virus (H1N1) relative to the 1968 virus (H3N2). Further, despite Mexico’s appreciably different past demographic trends (the largest cohorts in Mexico were born later than in the United States, i.e., in the early 1960s), excess mortality also peaks for the 1957 generation and yet again for the 1968 generation, without apparent connection between cohort size and peak mortality during the 2009 H1N1 pandemic and epidemic outbreak of the 2013–2014 influenza season.

The sheer size of the baby boom cohort could have had a role, not in directly making the members of this cohort more susceptible to death from influenza, but in making them much more likely to be infected with the H2N2 influenza virus very early in life than would have been the case if the cohort were smaller. It is possible that infections (priming with H2N2) at the time of birth or very early in life were more likely for the most numerous cohorts of the baby boom (1956 to 1961) than for the previous or following cohorts because of an increased rate of transmission. That increased fraction of the cohorts primed to a doubly heterosubtypic virus (i.e., H2N2 relative to the swine flu H1N1 virus) would have compounded its excess mortality in the 2009 and 2013–2014 influenza seasons.

The observation that susceptibility during pandemics seems to be elevated most profoundly for those primed early in life to doubly heterosubtypic viruses suggests that, contrary to the cross-protective immune responses elicited by early life exposure to antigenically similar viruses, priming by viruses with little-to-no antigenic overlap may result in responses that promote pathogenicity. This could be due to excessive proinflammatory responses mediated by T cells specific for conserved internal antigens and/or Fc-bearing cells that bind to cross-reactive, but nonneutralizing antibodies. Alternatively, early life infection with a virulent doubly heterosubtypic virus may compromise lung function (i.e., “scarring”) such that in the absence of cross-protective immunity, exposure to an antigenically unrelated virulent pandemic virus later in life causes exacerbated respiratory disease. These scenarios are not mutually exclusive, and we hope that our data will inform investigations to experimentally define the mechanism(s) at play. We recognize, however, that such experiments are challenging due to both difficulties in recapitulating the complex exposure histories of adults and difficulties associated with secondary infection of mice with influenza virus after a primary exposure.

In conclusion, we believe that there is an important missing piece in the puzzle that has been overlooked in most investigations concerning antigenic imprinting. Not only can antigenic imprinting lead to differential protection based on year of birth and exposure to antigenically related viruses in the past, but it can also lead to differential susceptibility to severe outcome from IAV infection as a result of mechanisms that appear to be distinct from those that explain differential protection. In all likelihood, attack rates were not especially high for the 1957 cohort in 2009 (see Fig. 7), yet the estimated CFRR was at its peak, suggesting the involvement of an aberrant immune reaction following infection, leading to severe outcomes or death. Our study does not allow for definitive conclusions to be drawn regarding the mechanisms which contribute to increased susceptibility. Importantly though, it provides a framework through which this phenomenon can be tested in animal models by identifying specific viral strains that can be used to recapitulate the exposure histories reported herein and define the biological basis of our observations.

Taken together, the findings reported herein firmly establish that exposure to a pandemic virus during the first years of life served as a susceptibility factor during later heterosubtypic influenza virus pandemics or intense seasonal outbreaks, indicating that it is not an isolated phenomenon. Developing experimental models to study the biological basis of this phenomenon will be essential to informing policy guidelines aimed at protecting high-risk groups during future pandemics.

## MATERIALS AND METHODS

Data on monthly mortality by age and underlying cause from October 1997 to July 2014 were obtained from the U.S. public-use data files of the National Center for Health Statistics (vital statistics data available online at https://www.cdc.gov/nchs/data_access/vitalstatsonline.htm) and from Mexico’s Instituto Nacional de Estadística y Geografía (INEGI) (http://www.beta.inegi.org.mx/proyectos/registros/vitales/mortalidad/default.html?init=2). We retrieved the monthly populations at risk by single year of age from the “1-year exposure to risk” tables published for the United States in the Human Mortality Database (http://www.mortality.org/) and the 1-year population size published for Mexico by the Consejo Nacional de Poblacion (CONAPO) (https://datos.gob.mx/busca/dataset/proyecciones-de-la-poblacion-de-mexico). In order to describe and compare mortality from influenza during pandemic and seasonal outbreaks, first we used raw pneumonia and influenza (P&I) counts, from which simple figures such as death counts can be readily and advantageously calculated for single ages. We used the “epidemic threshold” set by the Centers for Disease Control and Prevention (CDC) (Morbidity and Mortality Weekly Report https://www.cdc.gov/mmwr/preview/mmwrhtml/mm5929a2.htm and https://www.cdc.gov/mmwr/preview/mmwrhtml/mm6322a2.htm) to define the 2009 and 2013–2014 outbreaks in the United States, i.e., from October 2009 to January 2010, and from January to March 2014. No such analysis was readily available for Mexico, and thus, we defined the periods from daily counts of severe acute respiratory infections (SARI) deaths reported by Dávila-Torres et al. (18), i.e., from September 2009 to January 2010 and from December 2013 to March 2014. These selected dates are, to a certain extent, arbitrary and will lead to different numbers of deaths depending on the progression of the epidemic (the elderly are usually hit last yet have larger numbers of casualties). Therefore, several alternative specifications of outbreak periods were tested with no fundamental alterations in the results (see Text S1 in the supplemental material).

10.1128/mBio.02091-17.1 TEXT S1  Statistical and sensitivity analyses. Download TEXT S1, DOCX file, 0.02 MB. Copyright © 2018 Gagnon et al. 2018 Gagnon et al. This content is distributed under the terms of the Creative Commons Attribution 4.0 International license.

Since many of the deaths in the P&I category may be due to causes other than influenza (a pneumonia death may occur as the result of another source of infection unrelated to influenza), we also estimated “excess” mortality (strictly) from influenza with a Serfling model and a Serfling-inspired model that accounts for virus circulation, called here the “surveillance-Serfling” model (43) and presented below. We fit the surveillance-Serfling model to P&I deaths in the United States from October 1997 to July 2014, i.e., for the period during which both the indicators of influenza circulation and mortality data are available on a monthly basis. The monthly indicators such as influenza-like illness (ILI) or the percentage of respiratory specimens testing positive for influenza virus were taken from the World Health Organization FluNet database (http://www.who.int/influenza/gisrs_laboratory/flunet/en/). However, even though circulation data were available for Mexico, test applications of the surveillance-Serfling methods for this country proved unreliable, most likely because of population size issues, which are especially pronounced with single-year age data.

To favor specificity over sensitivity (25), we based our Serfling and surveillance-Serfling estimates on pneumonia and influenza (P&I) as the underlying causes of death rather than on all-cause mortality or other combinations, including respiratory and circulatory diseases, which are often involved in the etiology of influenza-related deaths. As a matter of fact, the aberrant pathological immune response, which we believe is implicated in the increased susceptibility to death of specific birth cohorts, is most easily revealed when P&I causes of death are selected to estimate influenza mortality. Given the purpose of this study, i.e., testing whether a birth during a specific (pandemic) year affects mortality from influenza during a subsequent pandemic later in life, it is necessary to tabulate yearly statistics. For the complete span of the 220 months under observation, i.e., from October 1997 to July 2014, we thus summarized the deaths caused by P&I by single-year age. Since virus circulation data are not available for single-year ages, we used instead virus circulation data by age group (0 to 4 years, 5 to 24 years, 25 to 64 years, and 65+ years).

### The Serfling model.

Serfling regression is a method widely used to estimate influenza mortality. It allows for the calculation of influenza mortality taking into account seasonal and secular mortality trends (44). The basic formulation of the model is:

$$\text{log}\left({\text{deaths}}_{a,t}\right)=\;\overset{5}{\underset{i=\;0}{\sum }}\beta_{i}t^{i}+\beta_{6}\text{sin}\left(\frac{2\pi t}{12}\right)+\beta_{7}\text{cos}\left(\frac{2\pi t}{12}\right)+\text{log}\left({\text{exposure}}_{a,t}\right)$$

where *a* is age in years (0, 1, 2, …, 100), *t* is the epidemic period (here from the 1997–1998 to the 2006–2007 seasons), the β's are parameters to estimate, *deaths*_*a*,*t*_ are the number of deaths, and *exposure*_*a*,*t*_ is the number of individuals at risk (population at risk). The Serfling model includes three principal components: $\left(\overset{5}{\underset{i=\;0}{\sum }}\beta_{i}t^{i}\right)$ is here a fifth degree polynomial that controls for secular trends in mortality, while the $\beta_{6}\text{sin}\left(\frac{2\pi t}{12}\right)+\beta_{7}\text{cos}\left(\frac{2\pi t}{12}\right)$ term captures influenza seasonality, and log(*exposure_a,t_*) accounts for changes in the distribution of the population by age (*a*) over time (*t*). This regression model is estimated accounting exclusively for summer months (May to October), when influenza virus is not supposed to circulate among the population of temperate regions. Influenza-related mortality is estimated as the difference between the observed monthly death counts recorded from P&I and the Serfling model-predicted death count.

In contrast to original applications of the Serfling method, which are based on linear regression models or Poisson distributions (44), we use a negative-binomial distribution, which accounts for overdispersion and allows for low-frequency-count data (45), which may indeed result from the single-year age classification used here. One advantage of the Serfling model is that it requires only death counts and the populations at risk by month and age. However, since it strongly relies on seasonal variations, this model may capture deaths unrelated to influenza that are also seasonal in nature, and it can produce incoherent estimates, such as negative numbers of deaths, as discussed by Nguyen and Noymer (25). Such instances indeed occurred in the Mexican data at young ages, which we alleviated by constraining the death count estimates to be equal or smaller than the observed P&I mortality, thereby “forcing” negative estimates to zero.

### The surveillance-Serfling model.

Provided that indicators of influenza activity are available and reliable, it is possible to use the surveillance-Serfling model, which may considerably improve the accuracy of the estimates (43, 44, 46, 47). In order to estimate mortality from influenza in the U.S. data, we used the following specification:

$$\text{log}\left({\text{deaths}}_{a,t}\right)=\;\overset{8}{\underset{i=\;0}{\sum }}\beta_{i}t^{i}+\beta_{9}\text{sin}\left(\frac{2\pi t}{12}\right)+\beta_{10}\text{cos}\left(\frac{2\pi t}{12}\right)+\beta_{11}\text{sin}\left(\frac{3\pi t}{12}\right)+\beta_{12}\;\cos\left(\frac{3\pi t}{12}\right)+\beta_{13}\text{sin}\left(\frac{4\pi t}{12}\right)+\beta_{14}\text{cos}\left(\frac{4\pi t}{12}\right)+\beta_{15}\;\sin\left(\frac{6\pi t}{12}\right)+\beta_{16}\text{cos}\left(\frac{6\pi t}{12}\right)+\beta_{17}\text{sin}\left(\frac{8\pi t}{12}\right)+\beta_{18}\text{cos}\left(\frac{8\pi t}{12}\right)+\beta_{19}\text{sin}\left(\frac{10\pi t}{12}\right)+\beta_{20}\text{cos}\left(\frac{10\pi t}{12}\right)+\text{log}\left({\text{exposure}}_{a,t}\right)+\beta_{21}{\text{PAN}}_{2009}+\beta_{22}{\text{PAN}}_{2013}+\beta_{23}{\text{ILI}}_{g,t}+\beta_{24}{\text{ILI}}_{g,\;t-1}$$

where *a* is age (0, 1, 2, …, 110), *t* is the monthly period (from October 1997 to July 2014), *deaths*_*a*,*t*_ are death counts (number of deaths), and *exposure*_*a*,*t*_ are the populations (number of individuals) at risk of age *a* at time *t*. As is the case for the traditional Serfling model, the sin and cos terms are harmonic terms that control for influenza seasonality, $\sum_{i=\;0}^{8}\beta_{i}t^{i}$ accounts for secular trends in mortality, and log(*exposure_a,t_*) tracks changes in the age structure of the population over time. Given age variations in mortality over time, specific components were retained for each age, based on their statistical significance (components with *P* values of >0.05 were rejected). The *PAN* terms are dummies that take the value of 1 during either the 2009 or 2013–2014 H1N1 outbreaks and of zero otherwise. The *ILI*_*g*,*t*_ term introduces the monthly incidence of influenza-like illnesses (ILI) for each age group (0 to 4 years, 5 to 24 years, 25 to 64 years, and 65+ years), including a 1-month lag term (*t* − 1), since an influenza death occurring during an index month may in fact result from an infection contracted the preceding month, particularly among the elderly individuals (42). For sensitivity analyses, we dropped the lag term in order to remove from the estimates the deaths arising from comorbidities and thus to sharpen our focus on deaths from influenza alone; the results were increased relative mortality among the younger generations relative to older generations (not shown here). As our earlier sex-specific analyses provided similar results for males and females, we decided to regroup sexes in order to improve model fit.

**FIG 6:**
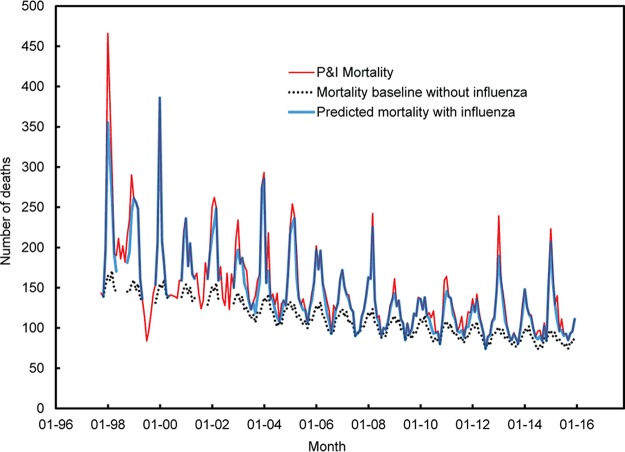
Surveillance-Serfling estimates of mortality per month from influenza for 80-year-old individuals. The number of deaths is shown on the *y* axis, and time (month-year) from January 1996 (01-96) to January 2016 (01-16) is shown on the *x* axis. The distance between the predicted number of deaths when there is influenza activity (blue line) and the number of deaths when there is no influenza activity (dotted black line) provides an estimate of deaths strictly due to influenza. The red line presents the raw death counts from P&I per month. Note that between 1997 and 2004, there were no data of ILI during the low influenza season, i.e., between June and September; the lines depicting the estimates of mortality with influenza activity (blue line) and without influenza activity (dotted black line) are thus interrupted during those periods, which are taken into account in this study.

We first fit the above model to deaths recorded in the P&I categories and then reran the model with the influenza activity terms, i.e., the *ILI* terms, set to zero to provide a baseline reflecting no influenza activity (and mortality). The difference between the estimation, including the *ILI* terms and the baseline is an estimate of mortality caused by influenza alone. For example, Fig. 6 shows mortality recorded within the P&I category for age 80 between October 1997 and December 2015 (in red), as well as mortality predicted by the surveillance-Serfling model (in blue) and a baseline with influenza activity terms set to zero (black dotted line); the distance between the blue line and the black dotted line provides an estimate of mortality from influenza.

Several other models were estimated with various influenza activity indicators, such as the distributions by subtype (H1N1, H3N2) as well as measures combining ILI incidence to subtype distributions (see reference 17 for a similar modeling strategy). Based on the Bayesian information criterion (BIC) (45), these models did not provide better fits than the model presented above. As argued elsewhere, the consideration of surveillance data may considerably improve the accuracy of the estimates in comparison with the usual Serfling model (43, 44, 46, 47), which is often plagued by negative estimates of the number of deaths from influenza, especially at older ages (25). It is worth noting that the surveillance-Serfling model used here produces very few such negative estimates. Those that occur are limited almost exclusively for summers, which are not taken into account in this analysis. The surveillance-based model also has the advantage of fitting data from all seasons, and not exclusively from the summer seasons, as does the traditional Serfling model; it thus produces higher mortality counts, which helps improve estimates for the single-year age data that are used in the present study. Yet, because we used 1-year age tabulations, some of the estimates provided here were not robust, especially for the young or the very old, where the number of deaths (numerator) and of individuals at risk (denominator) are very small. Given the purpose of our enquiry, i.e., to estimate mortality risk by yearly ages (and thus yearly birth cohorts), we presented smoothed curves along the raw estimates.

### Mortality rates, death rate ratios, and case fatality rate ratios.

**FIG 7:**
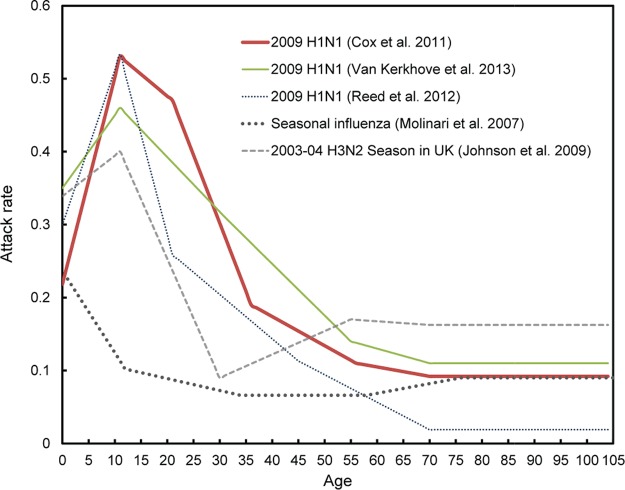
Estimated attack rates by age from influenza during the 2009 H1N1 pandemic and during seasonal outbreaks. The estimates reported by age groups by Cox et al. (30), Van Kerkhove et al. (49), Molinari et al. (53), and Reed et al. (55) were transformed into annualized values, using linear interpolation. Estimates for the 2003–2004 season were derived from Fig. 1 in reference 54 (Johnson et al.) by subtracting sera with titers above 40 in 2003 season from 2004 season and were also annualized using interpolation. Estimates for the elderly (65+) should be interpreted with caution in this figure (and in Fig. 5), as these attack rates pertain to very large age groups. Instead of interpolating for this age range, we used the nominal reported values after age 70 (i.e., 0.1) in the 2009 outbreak and after age 75 (0.09) in the seasonal outbreaks. No estimates of age-specific attack rates were readily available for the 2013–2014 H1N1 outbreak.

After estimating simple measures such as raw death counts from P&I mortality and from the above Serfling models, we estimated mortality rates due to influenza by single-year age, as well as mortality rate ratios by comparing mortality due to influenza during the 2009 and 2013–2014 H1N1 outbreaks to the average of the 1997–1999 to 2008–2009 influenza seasons. More precisely, for each month of each outbreak (seasonal or pandemic), we divided the number of deaths by the number of people of the same age and then took the monthly average over the whole duration of the outbreak (e.g., from October 2009 to January 2010 during the swine flu pandemic in the United States). Rate ratios by age were calculated by dividing the rate for each age by the monthly averaged death rates from the influenza seasons from 1997 to 2008. These age-specific rates were then displayed as cohorts to ease interpretation (Fig. 4). As mortality from influenza depends not only on susceptibility to severe outcomes following infection but also on the probability of being infected in the first place, we also used estimated attack rates from the literature. Dividing mortality rates by these attack rates, we estimated case fatality rates, as well as case fatality rate ratios (CFRRs), again by comparing the two H1N1 outbreaks of interest in this study to the average of the previous 11 years of seasonal epidemics. Attack rates by age groups were available for the 2009 pandemic in at least two meta-analyses (48, 49) and showed remarkable consistency between both studies and the countries examined. These estimates were obtained from serological assays; many of these assays subtracted the percentage of individuals with a titer above 40 before the outbreak from the corresponding percentage calculated after the outbreak to obtain “cumulative incidence” measures. For convenience, in Fig. 5 we used the estimates for six age groups (i.e., 0 to 4, 5 to 17, 18 to 24, 25 to 49, 50 to 64, and 65+ years) reported by Cox et al. (30), which we transformed into annualized values using linear interpolation in Fig. 7. Figure 7 also presents estimates from other studies, using broader age ranges.

Unfortunately, no such age-specific figures were readily available for the 2013–2014 outbreak and for the previous 11-year period that serves as a basis for comparison for this study. Most available estimates of attack rates by age date from the 20th century, and many go as far back as the 1960s. However, studies based on mathematical models and empirical results of the frequency of daily contacts by age (50) predict relatively robust distributions of the percentage of infected individuals by broadly defined age groups, which are associated with *R*_0_, the basic reproduction number (51, 52). These studies show that, excluding year-to-year fluctuations, on average, attack rates during seasonal outbreaks of influenza are highest for young children and adolescents, reaching values of around 20 to 30%, and fall relatively flat in adulthood with values between 5 and 10% for most of life. In Fig. 5, we used the empirical values reported by Molinari et al. (53), which follow this pattern (Fig. 7). Note, however, that emergence of a novel antigenic variant during a seasonal outbreak can also lead to disproportionate fractions of infected children among young children, such as occurred during the 2003–2004 H3N2 seasonal outbreak in the United Kingdom (54). This pattern at younger ages is expected, since the levels of preexisting immunity within the population are presumed to be low in the face of significant antigenic drift, as is the case during a pandemic (antigenic “shift”), although levels remain high at older ages. Since there were also some variations for the age-specific attack rates in 2009 reported in numerous studies, we provide sensitivity tests with variations in these attack rates, both for seasonal and pandemic outbreaks in the supplemental material (see Fig. S5 in the supplemental material).

## References

[1] Earn DJD, Dushoff J, Levin SA 2002 Ecology and evolution of the flu. Trends Ecol Evol 17:334–340.

[2] Miller MS, Palese P 2014 Peering into the crystal ball: influenza pandemics and vaccine efficacy. Cell 157:294–299. doi:10.1016/j.cell.2014.03.023. 24725400

[3] Taubenberger JK, Morens DM 2010 Influenza: the once and future pandemic. Public Health Rep 125(Suppl 3):16–26. doi:10.1177/00333549101250S305. PMC2862331 20568566

[4] Garten RJ, Davis CT, Russell CA, Shu B, Lindstrom S, Balish A, Sessions WM, Xu X, Skepner E, Deyde V, Okomo-Adhiambo M, Gubareva L, Barnes J, Smith CB, Emery SL, Hillman MJ, Rivailler P, Smagala J, de Graaf M, Burke DF, Fouchier RAM, Pappas C, Alpuche-Aranda CM, López-Gatell H, Olivera H, López I, Myers CA, Faix D, Blair PJ, Yu C, Keene KM, Dotson PD, Boxrud D, Sambol AR, Abid SH, St George K, Bannerman T, Moore AL, Stringer DJ, Blevins P, Demmler-Harrison GJ, Ginsberg M, Kriner P, Waterman S, Smole S, Guevara HF, Belongia EA, Clark PA, Beatrice ST, et al. 2009 Antigenic and genetic characteristics of swine-origin 2009 A(H1N1) influenza viruses circulating in humans. Science 325:197–201. doi:10.1126/science.1176225. 19465683 PMC3250984

[5] Palese P, Shaw M 2007 Orthomyxoviridae: the viruses and their replication, p 1647–1690. *In* Knipe DM, Griffin DE, Lamb RA, Straus SE, Howley PM, Martin MA, Roizman B (ed), Fields virology, 5th ed. Lippincott Williams & Wilkins, Philadelphia, PA.

[6] Gagnon A, Miller MS, Hallman SA, Bourbeau R, Herring DA, Earn DJ, Madrenas J 2013 Age-specific mortality during the 1918 influenza pandemic: unravelling the mystery of high young adult mortality. PLoS One 8:e69586. doi:10.1371/journal.pone.0069586. 23940526 PMC3734171

[7] Gagnon A, Acosta JE, Madrenas J, Miller MS 2015 Is antigenic sin always “original?” re-examining the evidence regarding circulation of a human H1 influenza virus immediately prior to the 1918 Spanish flu. PLoS Pathog 11:e1004615. doi:10.1371/journal.ppat.1004615. 25742615 PMC4351064

[8] Hallman SA 2015 The demographic links between the 1890 and 1918 influenza pandemics in Ontario. PhD dissertation University of Western Ontario, London, Ontario, Canada.

[9] Oeppen JE, Wilson C 2006 Epidemiological evidence for viral exposure in childhood as a risk-factor in subsequent influenza pandemics. Population Association of America, Los Angeles, CA.

[10] Dowdle WR 1999 Influenza A virus recycling revisited. Bull World Health Organ 77:820–828. 10593030 PMC2557748

[11] McCullers JA, Van De Velde L-A, Allison KJ, Branum KC, Webby RJ, Flynn PM 2010 Recipients of vaccine against the 1976 “swine flu” have enhanced neutralization responses to the 2009 novel H1N1 influenza virus. Clin Infect Dis 50:1487–1492. doi:10.1086/652441. 20415539 PMC2946351

[12] Xie H, Li X, Gao J, Lin Z, Jing X, Plant E, Zoueva O, Eichelberger MC, Ye Z 2011 Revisiting the 1976 “swine flu” vaccine clinical trials: cross-reactive hemagglutinin and neuraminidase antibodies and their role in protection against the 2009 H1N1 pandemic virus in mice. Clin Infect Dis 53:1179–1187. doi:10.1093/cid/cir693. 21976461

[13] Miller MS, Tsibane T, Krammer F, Hai R, Rahmat S, Basler CF, Palese P 2013 1976 and 2009 H1N1 influenza virus vaccines boost anti-hemagglutinin stalk antibodies in humans. J Infect Dis 207:98–105. doi:10.1093/infdis/jis652. 23087428 PMC3523798

[14] Ellebedy AH, Krammer F, Li G-M, Miller MS, Chiu C, Wrammert J, Chang CY, Davis CW, McCausland M, Elbein R, Edupuganti S, Spearman P, Andrews SF, Wilson PC, García-Sastre A, Mulligan MJ, Mehta AK, Palese P, Ahmed R 2014 Induction of broadly cross-reactive antibody responses to the influenza HA stem region following H5N1 vaccination in humans. Proc Natl Acad Sci U S A 111:13133–13138. doi:10.1073/pnas.1414070111. 25157133 PMC4246941

[15] Miller MS, Gardner TJ, Krammer F, Aguado LC, Tortorella D, Basler CF, Palese P 2013 Neutralizing antibodies against previously encountered influenza virus strains increase over time: a longitudinal analysis. Sci Transl Med 5:198ra107. doi:10.1126/scitranslmed.3006637. PMC4091683 23946196

[16] Nachbagauer R, Wohlbold TJ, Hirsh A, Hai R, Sjursen H, Palese P, Cox RJ, Krammer F 2014 Induction of broadly reactive anti-hemagglutinin stalk antibodies by an H5N1 vaccine in humans. J Virol 88:13260–13268. doi:10.1128/JVI.02133-14. 25210189 PMC4249097

[17] Borja-Aburto VH, Chowell G, Viboud C, Simonsen L, Miller MA, Grajales-Muñiz C, González-Bonilla CR, Diaz-Quiñonez JA, Echevarría-Zuno S 2012 Epidemiological characterization of a fourth wave of pandemic A/H1N1 influenza in Mexico, winter 2011-2012: age shift and severity. Arch Med Res 43:563–570. doi:10.1016/j.arcmed.2012.09.005. 23079035 PMC3545473

[18] Dávila-Torres J, Chowell G, Borja-Aburto VH, Viboud C, Grajalez-Muñiz C, Miller MA 2015 Intense seasonal A/H1N1 influenza in Mexico, winter 2013-2014. Arch Med Res 46:63–70. doi:10.1016/j.arcmed.2014.11.005. 25446618

[19] Worobey M, Han G-Z, Rambaut A 2014 Genesis and pathogenesis of the 1918 pandemic H1N1 influenza A virus. Proc Natl Acad Sci U S A 111:8107–8112. doi:10.1073/pnas.1324197111. 24778238 PMC4050607

[20] Shanks GD, Brundage JF 2012 Pathogenic responses among young adults during the 1918 influenza pandemic. Emerg Infect Dis 18:201–207. doi:10.3201/eid1802.102042. 22306191 PMC3310443

[21] Ma J, Dushoff J, Earn DJD 2011 Age-specific mortality risk from pandemic influenza. J Theor Biol 288:29–34. doi:10.1016/j.jtbi.2011.08.003. 21856313

[22] Almond D 2006 Is the 1918 influenza pandemic over? Long-term effects of in utero influenza exposure in the post-1940 U.S. population. J Polit Econ 114:672–712. doi:10.1086/507154.

[23] Shrestha SS, Swerdlow DL, Borse RH, Prabhu VS, Finelli L, Atkins CY, Owusu-Edusei K, Bell B, Mead PS, Biggerstaff M, Brammer L, Davidson H, Jernigan D, Jhung MA, Kamimoto LA, Merlin TL, Nowell M, Redd SC, Reed C, Schuchat A, Meltzer MI 2011 Estimating the burden of 2009 pandemic influenza A (H1N1) in the United States (April 2009-April 2010). Clin Infect Dis 52(Suppl 1):S75–S82. doi:10.1093/cid/ciq012. 21342903

[24] Serfling RE 1963 Methods for current statistical analysis of excess pneumonia-influenza deaths. Public Health Rep 78:494–506. doi:10.2307/4591848. 19316455 PMC1915276

[25] Nguyen AM, Noymer A 2013 Influenza mortality in the United States, 2009 pandemic: burden, timing and age distribution. PLoS One 8:e64198. doi:10.1371/journal.pone.0064198. 23717567 PMC3661470

[26] Li Y, Myers JL, Bostick DL, Sullivan CB, Madara J, Linderman SL, Liu Q, Carter DM, Wrammert J, Esposito S, Principi N, Plotkin JB, Ross TM, Ahmed R, Wilson PC, Hensley SE 2013 Immune history shapes specificity of pandemic H1N1 influenza antibody responses. J Exp Med 210:1493–1500. doi:10.1084/jem.20130212. 23857983 PMC3727314

[27] Linderman SL, Chambers BS, Zost SJ, Parkhouse K, Li Y, Herrmann C, Ellebedy AH, Carter DM, Andrews SF, Zheng N-Y, Huang M, Huang Y, Strauss D, Shaz BH, Hodinka RL, Reyes-Terán G, Ross TM, Wilson PC, Ahmed R, Bloom JD, Hensley SE 2014 Potential antigenic explanation for atypical H1N1 infections among middle-aged adults during the 2013-2014 influenza season. Proc Natl Acad Sci U S A 111:15798–15803. doi:10.1073/pnas.1409171111. 25331901 PMC4226110

[28] Simonsen L, Spreeuwenberg P, Lustig R, Taylor RJ, Fleming DM, Kroneman M, Van Kerkhove MD, Mounts AW, Paget WJ, GLaMOR Collaborating Teams 2013 Global mortality estimates for the 2009 influenza pandemic from the GLaMOR project: a modeling study. PLoS Med 10:e1001558. doi:10.1371/journal.pmed.1001558. 24302890 PMC3841239

[29] Kilbourne ED 2006 Influenza pandemics of the 20th century. Emerg Infect Dis 12:9–14. doi:10.3201/eid1201.051254. 16494710 PMC3291411

[30] Cox CM, Goodin K, Fisher E, Dawood FS, Hamilton JJ, Leparc GF, Gray M, Nelson L, Borse RH, Singleton JA, Reed C, Balish AL, Katz JM, Hopkins RS, Fry AM 2011 Prevalence of 2009 pandemic influenza A (H1N1) virus antibodies, Tampa Bay Florida—November-December, 2009. PLoS One 6:e29301. doi:10.1371/journal.pone.0029301. 22206008 PMC3243696

[31] Earn DJD, He D, Loeb MB, Fonseca K, Lee BE, Dushoff J 2012 Effects of school closure on incidence of pandemic influenza in Alberta, Canada. Ann Intern Med 156:173–181. doi:10.7326/0003-4819-156-3-201202070-00005. 22312137

[32] Ikonen N, Strengell M, Kinnunen L, Osterlund P, Pirhonen J, Broman M, Davidkin I, Ziegler T, Julkunen I 2010 High frequency of cross-reacting antibodies against 2009 pandemic influenza A(H1N1) virus among the elderly in Finland. Euro Surveill 15(5):pii=19478. 20144443

[33] Fisman DN, Savage R, Gubbay J, Achonu C, Akwar H, Farrell DJ, Crowcroft NS, Jackson P 2009 Older age and a reduced likelihood of 2009 H1N1 virus infection. N Engl J Med 361:2000–2001. doi:10.1056/NEJMc0907256. 19907052

[34] Jacobs JH, Archer BN, Baker MG, Cowling BJ, Heffernan RT, Mercer G, Uez O, Hanshaoworakul W, Viboud C, Schwartz J, Tchetgen Tchetgen E, Lipsitch M 2012 Searching for sharp drops in the incidence of pandemic A/H1N1 influenza by single year of age. PLoS One 7:e42328. doi:10.1371/journal.pone.0042328. 22876316 PMC3410923

[35] Medina RA, Stertz S, Manicassamy B, Zimmermann P, Sun X, Albrecht RA, Uusi-Kerttula H, Zagordi O, Belshe RB, Frey SE, Tumpey TM, García-Sastre A 2013 Glycosylations in the globular head of the hemagglutinin protein modulate the virulence and antigenic properties of the H1N1 influenza viruses. Sci Transl Med 5:187ra70. doi:10.1126/scitranslmed.3005996. PMC3940933 23720581

[36] Cobey S, Hensley SE 2017 Immune history and influenza virus susceptibility. Curr Opin Virol 22:105–111. doi:10.1016/j.coviro.2016.12.004. 28088686 PMC5467731

[37] Reber A, Katz J 2013 Immunological assessment of influenza vaccines and immune correlates of protection. Expert Rev Vaccines 12:519–536. doi:10.1586/erv.13.35. 23659300 PMC9002926

[38] Girard MP, Tam JS, Assossou OM, Kieny MP 2010 The 2009 A (H1N1) influenza virus pandemic: a review. Vaccine 28:4895–4902. doi:10.1016/j.vaccine.2010.05.031. 20553769

[39] Krammer F, Palese P 2015 Advances in the development of influenza virus vaccines. Nat Rev Drug Discov 14:167–182. doi:10.1038/nrd4529. 25722244

[40] Gostic KM, Ambrose M, Worobey M, Lloyd-Smith JO 2016 Potent protection against H5N1 and H7N9 influenza via childhood hemagglutinin imprinting. Science 354:722–726. doi:10.1126/science.aag1322. 27846599 PMC5134739

[41] Rau R, Bohk C, Muszynska M, Vaupel J 2013 Rates of mortality improvement on the Lexis surface: visualizing age-, period-, and cohort-effects. Population Association of America, Washington, DC.

[42] Acosta E, Miller M, Hallman S, Bourbeau R, Herring DA, Dillon L, Earn D, Madrenas J, Gagnon A 2016 Determinants of influenza mortality trends: early-life exposure to influenza and age-period-cohort analysis of influenza mortality in U.S. 1959–2012. Population Association of America, Washington, DC.

[43] Lemaitre M, Carrat F, Rey G, Miller M, Simonsen L, Viboud C 2012 Mortality burden of the 2009 A/H1N1 influenza pandemic in France: comparison to seasonal influenza and the A/H3N2 pandemic. PLoS One 7:e45051. doi:10.1371/journal.pone.0045051. 23028756 PMC3447811

[44] Thompson WW, Weintraub E, Dhankhar P, Cheng P-Y, Brammer L, Meltzer MI, Bresee JS, Shay DK 2009 Estimates of US influenza-associated deaths made using four different methods. Influenza Other Respir Viruses 3:37–49. doi:10.1111/j.1750-2659.2009.00073.x. 19453440 PMC4986622

[45] Hilbe JM 2011 Negative binomial regression, 2nd ed Cambridge University Press, Cambridge, United Kingdom.

[46] Thompson WW, Shay DK, Weintraub E, Brammer L, Cox N, Anderson LJ, Fukuda K 2003 Mortality associated with influenza and respiratory syncytial virus in the United States. JAMA 289:179–186. doi:10.1001/jama.289.2.179. 12517228

[47] Simonsen L, Viboud C, Taylor RJ, Miller MA 2011 The epidemiology of influenza and its control, p 27–54. *In* Rappuoli R, Del Giudice G (ed), Influenza vaccines for the future. Springer, Basel, Switzerland.

[48] Jayasundara K, Soobiah C, Thommes E, Tricco AC, Chit A 2014 Natural attack rate of influenza in unvaccinated children and adults: a meta-regression analysis. BMC Infect Dis 14:670. doi:10.1186/s12879-014-0670-5. 25495228 PMC4272519

[49] Van Kerkhove MD, Hirve S, Koukounari A, Mounts AW, H1N1pdm Serology Working Group 2013 Estimating age-specific cumulative incidence for the 2009 influenza pandemic: a meta-analysis of A(H1N1)pdm09 serological studies from 19 countries. Influenza Other Respir Viruses 7:872–886. doi:10.1111/irv.12074. 23331969 PMC5781221

[50] Mossong J, Hens N, Jit M, Beutels P, Auranen K, Mikolajczyk R, Massari M, Salmaso S, Tomba GS, Wallinga J, Heijne J, Sadkowska-Todys M, Rosinska M, Edmunds WJ 2008 Social contacts and mixing patterns relevant to the spread of infectious diseases. PLoS Med 5:e74. doi:10.1371/journal.pmed.0050074. 18366252 PMC2270306

[51] Skowronski DM, Moser FS, Janjua NZ, Davoudi B, English KM, Purych D, Petric M, Pourbohloul B 2013 H3N2v and other influenza epidemic risk based on age-specific estimates of sero-protection and contact network interactions. PLoS One 8:e54015. doi:10.1371/journal.pone.0054015. 23326561 PMC3543419

[52] Gambhir M, Swerdlow DL, Finelli L, Van Kerkhove MD, Biggerstaff M, Cauchemez S, Ferguson NM 2013 Multiple contributory factors to the age distribution of disease cases: a modeling study in the context of influenza A(H3N2v). Clin Infect Dis 57(Suppl 1):S23–S27. doi:10.1093/cid/cit298. 23794728 PMC3689451

[53] Molinari N-AM, Ortega-Sanchez IR, Messonnier ML, Thompson WW, Wortley PM, Weintraub E, Bridges CB 2007 The annual impact of seasonal influenza in the US: measuring disease burden and costs. Vaccine 25:5086–5096. doi:10.1016/j.vaccine.2007.03.046. 17544181

[54] Johnson BF, Wilson LE, Ellis J, Elliot AJ, Barclay WS, Pebody RG, McMenamin J, Fleming DM, Zambon MC 2009 Fatal cases of influenza A in childhood. PLoS One 4:e7671. doi:10.1371/journal.pone.0007671. 19876396 PMC2764845

[55] Reed C, Katz JM, Hancock K, Balish A, Fry AM, H1N1 Serosurvey Working Group. 2012 Prevalence of seropositivity to pandemic influenza A/H1N1 virus in the United States following the 2009 pandemic. PLoS One 7:e48187. doi:10.1371/journal.pone.0048187. 23118949 PMC3485186

