# Supplementary figures and images for: Pandemic Paradox: Early Life H2N2 Pandemic Influenza Infection Enhanced Susceptibility to Death during the 2009 H1N1 Pandemic
Source: mBio. 2018 Jan 16;9(1):e02091-17. doi: 10.1128/mBio.02091-17 (PMC5770550; doi:10.1128/mBio.02091-17)

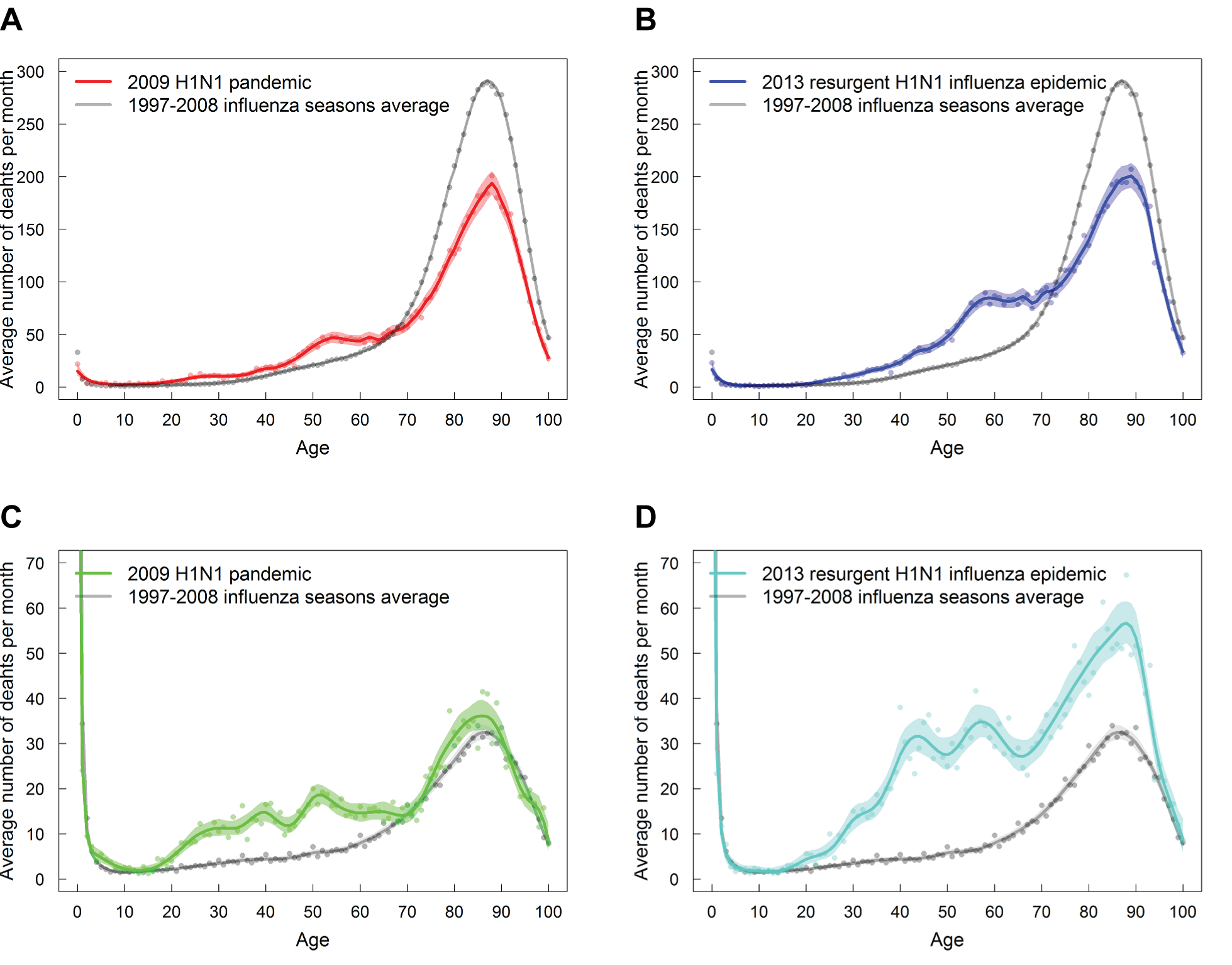

Supplement: FIG S1 [file mbo001183684sf1.tif]

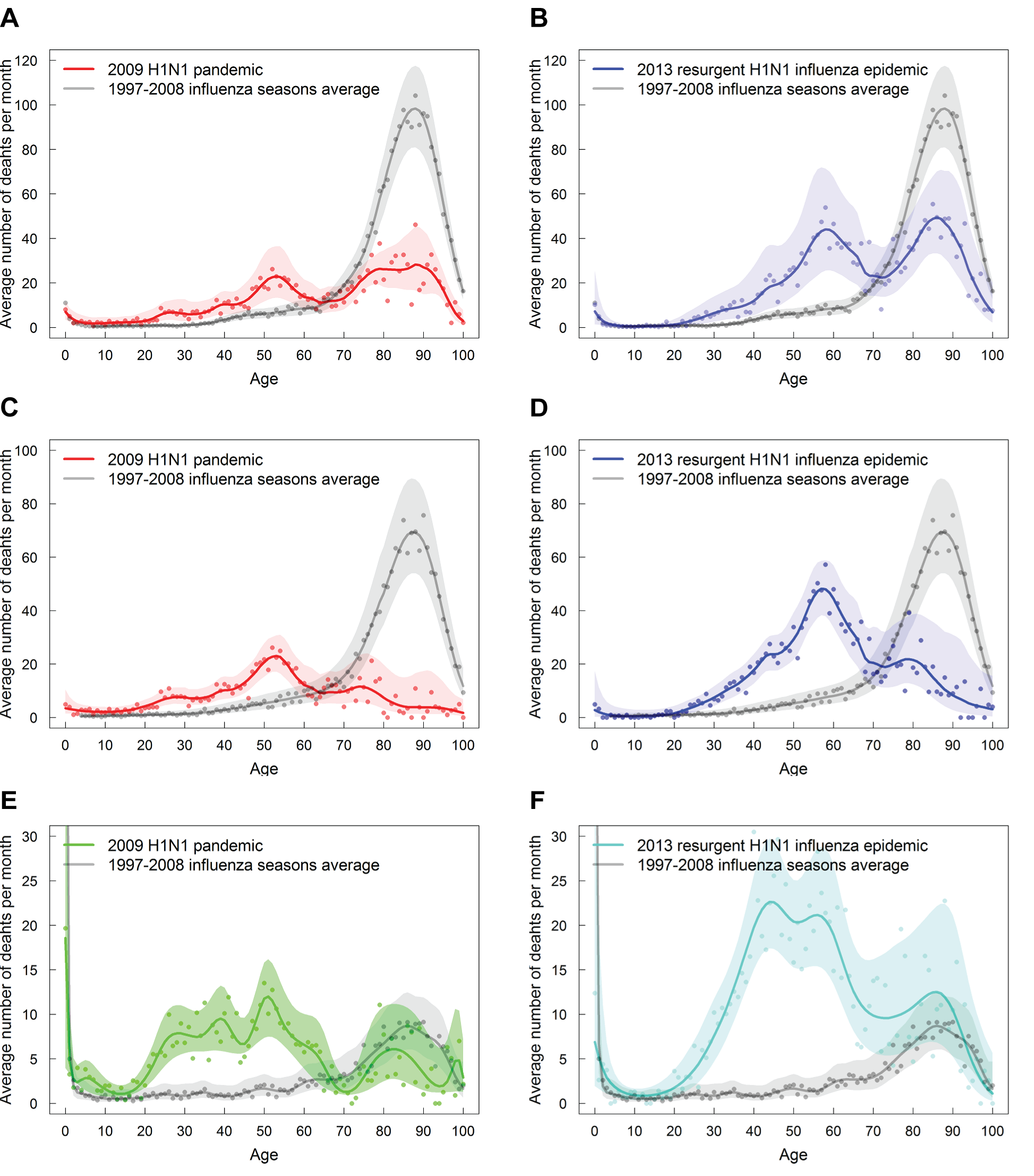

Supplement: FIG S2 [file mbo001183684sf2.tif]

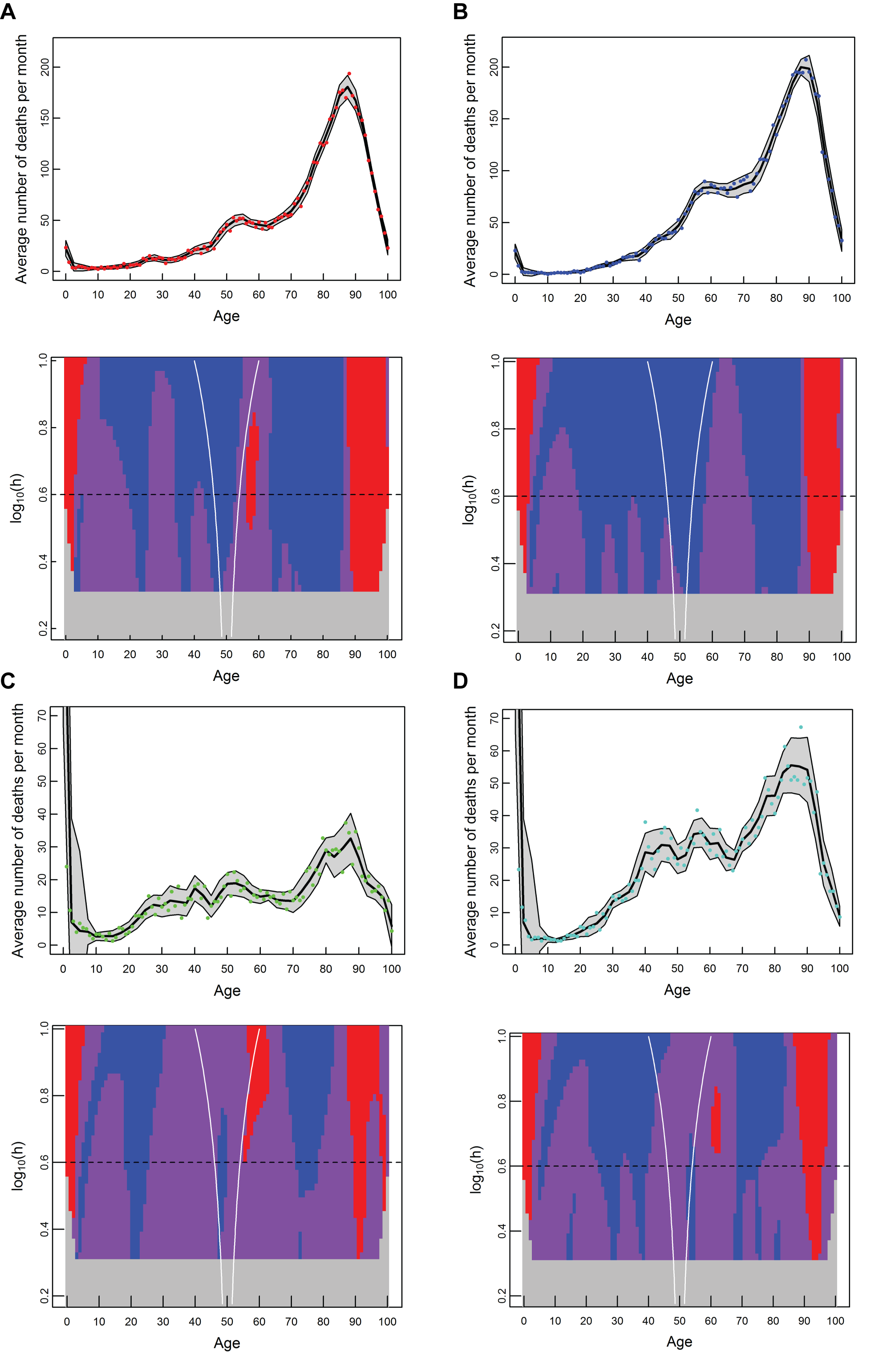

Supplement: FIG S3 [file mbo001183684sf3.tif]

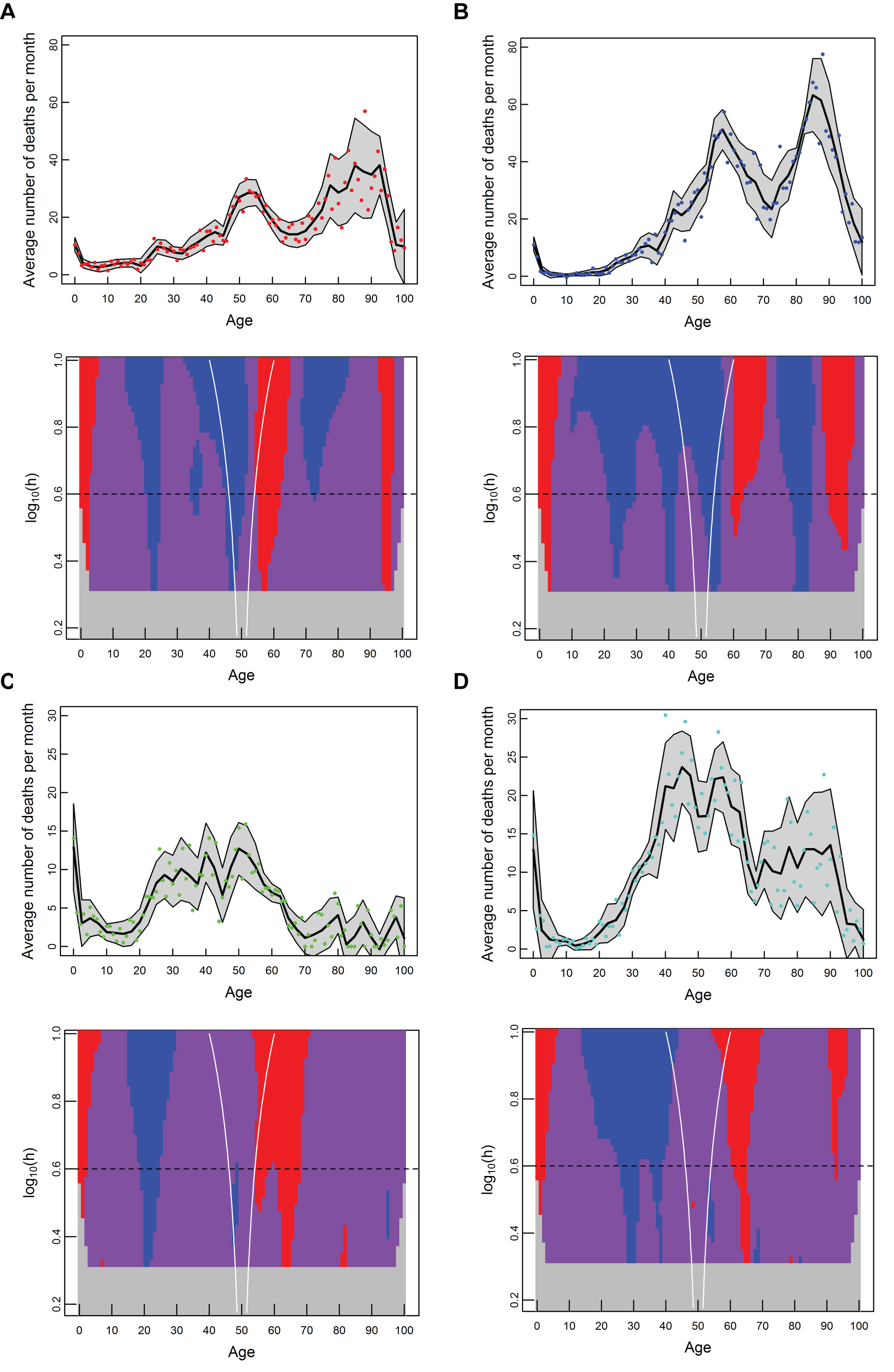

Supplement: FIG S4 [file mbo001183684sf4.tif]

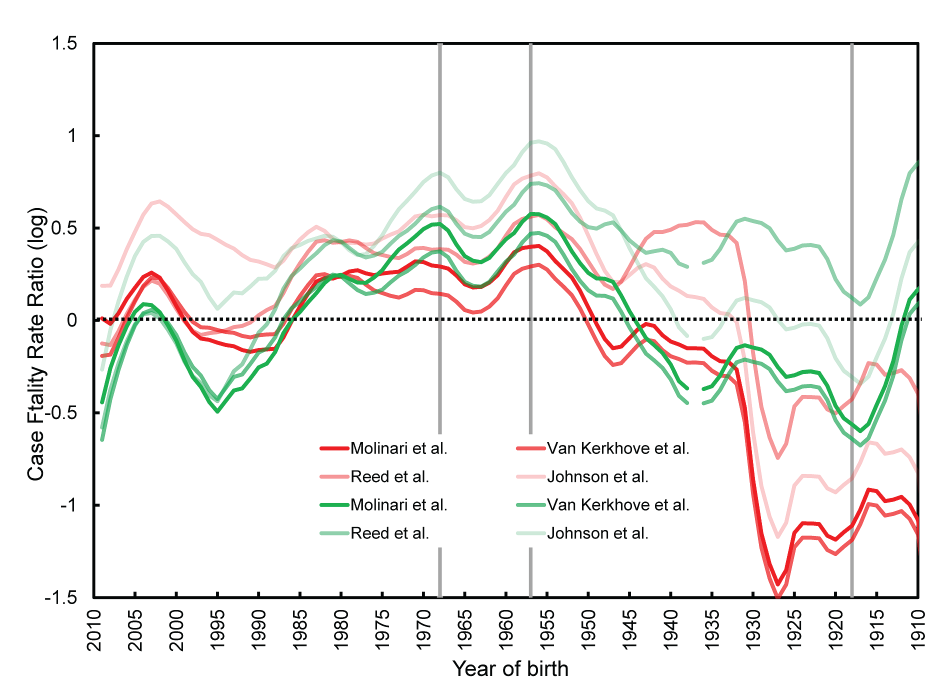

Supplement: FIG S5 [file mbo001183684sf5.tif]

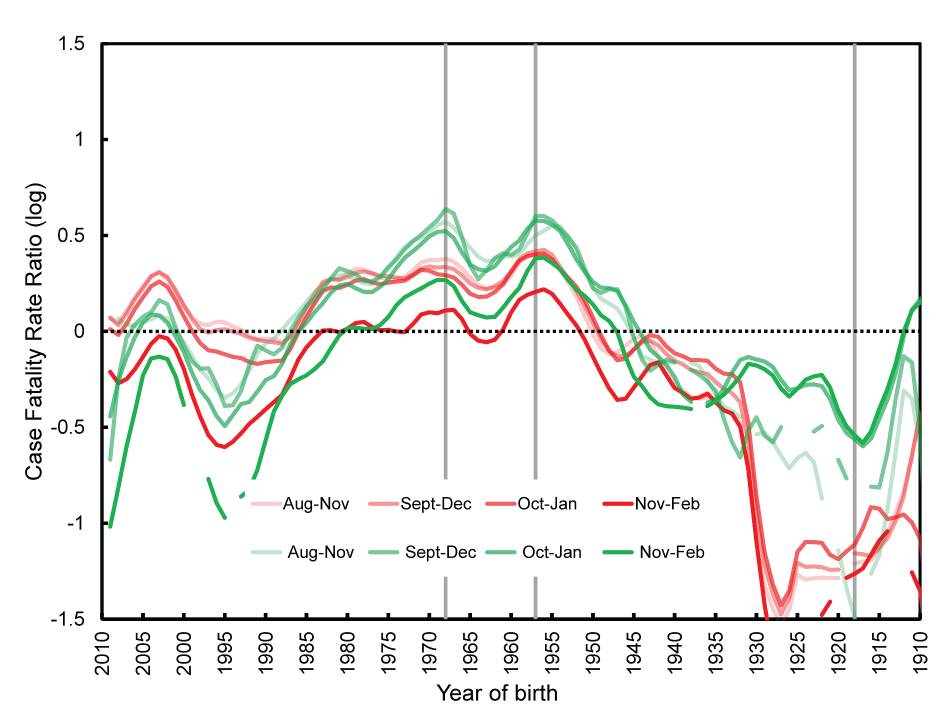

Supplement: FIG S6 [file mbo001183684sf6.tif]
