# Supplementary material for: Pandemic Paradox: Early Life H2N2 Pandemic Influenza Infection Enhanced Susceptibility to Death during the 2009 H1N1 Pandemic
Source: mBio. 2018 Jan 16;9(1):e02091-17. doi: 10.1128/mBio.02091-17 (PMC5770550; doi:10.1128/mBio.02091-17)
Supplement: TEXT S1 [file mbo001183684s1.docx]

**SUPPLEMENTARY MATERIAL**

**STATISTICAL AND SENSITIVITY ANALYSES**

**Confidence intervals**

In this section we present death counts estimated by a flexible smoothing method based on P-splines (1). This approach allowed us to compute confidence intervals for death count estimates, and thus to assess the precision of our results. Although P-spline-smoothed lines may not look fundamentally different from those produced by other smoothing techniques such as lowess, P-splines offer further desirable (asymptotic and practical) properties over competing smoothers (1). Of particular interest here is the P-splines’ advantage of accounting for the size of the population at risk in the computation of confidence intervals for death count estimates.

In a nutshell, the P-spline method is a nonparametric approach that combines the concepts of B-spline and penalized likelihood. B-splines provide flexibility, which leads to an accurate fit of the data, while the penalty acts on the coefficients of adjacent B-splines to ensure that the resulting fit behaves smoothly. In the context of mortality data, the P-spline method can be adapted to model death counts within a Poisson regression setting, where the penalty occurs as part of a penalized Poisson likelihood function (see (2) for further details). Conveniently, the statistical programming environment R (3) features a package called “MortalitySmooth” (4) that performs smoothing of Poisson death counts with P-splines over a given range of ages and/or calendar years. Confidence intervals are readily available with the package because they are actually borrowed directly from classical regression theory (i.e., confidence intervals are constructed using the parameters’ standard errors obtained from the “hat” matrix). The confidence intervals for estimated death counts, for instance, thus account for the size of the population at risk of death.

In the following analyses, P-splines were first employed to smooth raw mortality data, using the “Mort1Dsmooth” function from the MortalitySmooth package, which can accommodate for over-dispersion (relative to a Poisson model). We opted for the argument “method=2” of “Mort1Dsmooth” to select the smoothing parameter, λ, according to the AIC (rather than selecting the value of λ subjectively). Figure S1 presents the number of deaths from P&I mortality during the 2009 and 2013-14 outbreaks, along with the seasonal average figures. It is clear that mortality from influenza were at levels well above usual statistical significance thresholds for adults < 65 during the 2009 pandemic in both the US and Mexico; the same was true during the 2013-14 epidemic in the US, and for all adults aged 20+ in Mexico.

Second, we present smoothed estimates of deaths counts based on the Surveillance-Serfling and the traditional Serfling models, again using Poisson P-splines. The computation of confidence intervals for these estimates is not straightforward, since both the variability resulting from the application of the Serfling and the P-splines models need to be factored in and accounted for. As a partial and conservative solution, we added the confidence intervals from the two models. We first derived the confidence intervals from the Serfling models, and then smoothed the upper and lower bounds of these intervals in order to obtain the final confidence intervals; in other words, the upper bound of the former (the “upper-upper” bound) and the lower bound of the latter (the “lower-lower”) served as a basis to build the confidence intervals that are presented in Figure S2. Again, in order to avoid over-saturating the figure, values for the 2009 pandemic are presented separately from those of the 2013-14 epidemics. As is immediately clear, the confidences intervals derived from the Serfling models are larger than those derived from raw P&I mortality data; this is because of the variability intrinsic to the application of the Serfling method. This is especially true for the Surveillance-Serfling method, which, if allegedly preferable to the traditional Serfling method, uses many parameters, leading to somewhat larger confidence intervals in panels A and B for the US data. Applying the more frugal traditional Serfling method to the same US data (Panel C and D) and, to a lesser extent, to the Mexican data (Panel E and F), leads to narrower confidence intervals. Taken together, panels from Figures S1and S2 provide strong evidence that patterns of excess mortality among adults presented in the result section of this paper are genuine and not the result of chance occurrence during specific influenza outbreaks. Yet, they provide little evidence that *specific* cohorts were at an increased risk of death due to exposure early in life to specific pandemic influenza strains. The “SiZer plots” of the next section help to provide support for this claim.

**SiZer plots**

While smoothing techniques such as “lowess” are a useful tool for finding regularities or specific features in data, such as a region of increase or decrease, local maxima or minima, it also has the potential drawback of highlighting spurious features. “SIgnificant ZERo crossing of derivatives” (SiZer) is a method allowing an inspection of the data at a wide range of smoothing bandwidths. Similar to the P-Splines method used above, it thus alleviates the traditional problem of choosing between bandwidth or smoothing parameters and helping to decipher whether features noticed at specific bandwidths are likely to be genuine features, or rather the result of natural (random) variation (5). It is well known for instance that small bandwidths may result in undersmoothing, with low bias but large variance, while large bandwidth may result in over-smoothing, with low variance but large bias. Features seen across bandwidths, on the other hand, would point to important and genuine underlying features (6).

More precisely, a genuine excess mortality of those born at the time of the of the 1957 pandemic should translate, over a large range of bandwidths, into a significant increase of mortality in the ages leading up to age 52, followed by a peak, and then by a significant decrease. In other words, the first derivative of the smoothed curve should be significantly positive in the ages leading up to age 52 (i.e., the confidence interval of the first derivative contains only positive values), possibly zero around that age (the CI contains zero) and then significantly negative thereafter (the CI contains only negative values). In principle, several methods of smoothing could be used (such as P-spline, used earlier here) to build SiZer plot analyses. For convenience, we used locally weighted polynomial regressions implemented in the SiZer package version 0.1–4.0 (7) in the statistical software R (3).

Figure S3 presents P&I death counts and a smooth of these deaths counts, using polynomial regressions, along with the confidence intervals. We chose a Gaussian kernel with a polynomial degree of 2, over a range of bandwidth from 1.5 to 10 years in the SiZer graphs of each panel. Panels A and C (i.e., referring to mortality in the US and Mexico in 2009) point, over a large number of bandwidths, to an increase (blue) of mortality prior to age 52, followed by a plateau (purple) and a drop (red), thus providing support for a local peak at age 52. On the other hand, the peak for the same cohorts (born around 1957) during the 2013-14 epidemic, i.e., at age around 56 years, covers much fewer bandwidths for Mexico and is not seen for the US. Additionally, there is no evidence for a local peak for the 1968 generation, in both the US and Mexico.

We also applied the same methodology to estimates of deaths from Serfling models (Figure S4). Note however, that Figure S4 must be interpreted with caution because it does not factor in variability from the Serfling models, as was done in Figure 9. Nevertheless, assuming that estimates from these models are largely an accurate portrait of the true values, the evidence for a local peak at the age of 52 during the 2009 pandemic and at age 56-57 during the 2013-14 is particularly clear. Owing to smaller sample size and thus larger random fluctuations, the evidence is much weaker for the Mexican data. Yet, we note that mortality is already high in the ages leading up to age 52 in Mexico, which largely accounts for the fact that the blue area depicting a significant increase prior to that age is rather restricted. As expected, death counts also decrease in Mexico after age 50-55 (red).

**Sensitivity analyses**

Results reported in Figure 5 depend on attack rates provided in Figure 7. In the absence of direct serological measures, the age-specific immunological profiles could have been estimated from the data on clinical attack rates. Yet, although this data offers the advantage of being available on a weekly basis, allowing for a tracking of the epidemic practically in “real time”, it has the disadvantage of not capturing asymptomatic or mild cases (without recorded ILI symptoms), leading to important underestimations, which may be age-specific. We thus used estimates obtained from serological assays because we seek to identify groups presenting high susceptibility to severe outcomes among all those infected, and not only among those with severe enough infections to require hospitalization or to show up in hospital or clinical tabulations. For convenience, we used the traditional Serfling method for both U.S. and Mexican data in the sensitivity analyses.

Figure S5 presents CFRRs using alternative specifications of attack rates for the 1997-2008 seasonal averages. Although there are noticeable variations, there are no fundamental changes relative to Figure 5, and the general picture remains the same.

As another sensitivity analysis, we wished to specify alternative periods to estimate CFRRs the 2009 pandemic, using a four-month sliding window, beginning with August-November 2009 and ending with November 2009-February 2010 (Figure S6). To improve comparability, we again used the exact same method (i.e., the traditional Serfling model) for the two countries under observation and the same periods. The similarity between the two countries is yet again striking. As expected, the CFRRs are higher for both the US and Mexico at the beginning of the outbreak and decrease as the pandemic progresses. Nevertheless, the overall pattern is not fundamentally altered relative to Figure 5, with noticeable peaks CFRRs for the 1957 and, to a lesser extent, for the 1968 cohorts.

**SUPPLEMENTARY FIGURE LEGENDS**

**Fig S1: Average number of deaths per month from pneumonia and influenza (P&I) during the 2009 influenza pandemic, the 2013-14 resurgent H1N1 influenza epidemic and during the 1997-2008 influenza seasons in the US (A and B) and Mexico (C and D).** Smoothed lines and 95% confidence intervals were obtained by P-Spline smoothing based on penalized Poisson likelihood.

**Fig. S2. Average number of deaths per months estimated from Surveillance-Serfling (A and B) and Serfling models (C and D), for the US (A-D) and Mexico (E and F).** Poisson P-Splines were used to smooth death count estimates. Shaded areas depict 95% confidence intervals, which account for variation in both Serfling and P-splines models.

**Fig. S3. Average number of deaths per month from P&I and SiZer plots of the first derivatives of polynomial smooths of these estimates, A and B) US and C and D) Mexico, A, C) 2009 and B, D) 2013-14**. The age-specific death counts used to build polynomial smooths in the upper figures are plotted as colored circles, along with the 95% confidence intervals in gray. In the SiZer plots (lower figures), the color scheme is blue in the locations where the curve is significantly increasing, red when it is significantly decreasing and purple where it cannot be declared whether it is increasing or decreasing. Light grey represents areas where the data are too sparse to make statement about significance (the polynomial weights rely on too few points, i.e., the effective sample size in the window is less than 5). The horizontal line at h=log (0.6) = 4 in the SiZer plots represents a bandwidth of 4, used to create the polynomial smooth in the upper figure of each panel. plots. The white curves in the SiZer plots provide a sense of the window widths for each bandwidth; the horizontal distance between the two lines is 2h (i.e., +2 standard deviations of the Gaussian kernel).

**Fig. S4. Average number of deaths per month estimated from Surveillance-Serfling and Serfling models, and SiZer plots of the first derivatives of polynomial smooths of these estimates, US and Mexico, 2009 and 2013-14**. The age-specific death counts used to build polynomial smooths in the upper figures are plotted as colored circles, along with the 95% confidence intervals in gray. In the SiZer plots (lower figures), the color scheme is blue in the locations where the curve is significantly increasing, red when it is significantly decreasing and purple where it cannot be declared whether it is increasing or decreasing. Light grey represents areas where the data are too sparse to make statement about significance (the polynomial weights rely on too few points, i.e., the effective sample size in the window is less than 5). The horizontal line at h=log (0.6) = 4 in the SiZer plots represents a bandwidth of 4, used to create the polynomial smooth in the upper figure of each panel. The white curves in the SiZer plots provide a sense of the window widths for each bandwidth; the horizontal distance between the two lines is 2h (i.e., +2 standard deviations of the Gaussian kernel).

**Fig. S5. Case fatality rate ratios (CFRR) of influenza mortality during the 2009 H1N1 influenza pandemic using alternative attack rates.** Ratios of case fatality rates are relative to the average of values for influenza seasons from 1998-2008. For both the 2009 pandemic and the flu seasons, age-specific case fatality rates were obtained by dividing death rates by the attack rates provided in Figure 7 for each age. The log (base 10) is used on the Y-axis in order to preserve symmetry about a case fatality rate ratio of 1 (i.e., the zero-line on a log scale). Shaded vertical bar mark the 1918, 1957 and 1968 influenza epidemics. Only smoothed values are presented to ease the reading of the figure.

**Fig. S6. Case fatality rate ratios (CFRR) of influenza mortality during the 2009 H1N1 influenza pandemic using alternative pandemic period definitions.** Ratios of case fatality rates are relative to the average of values for influenza seasons from 1998-2008. For both the 2009 pandemic and the flu seasons, age-specific case fatality rates were obtained by dividing death rates by the attack rates provided in Figure 7 for each age. The log (base 10) is used on the Y-axis in order to preserve symmetry about a case fatality rate ratio of 1 (i.e., the zero-line on a log scale). Shaded vertical bar mark the 1918, 1957 and 1968 influenza epidemics. Only smoothed values are presented to ease the reading of the figure.

**REFERENCES**

1. **Eilers P**, **Marx B**. 1996. Flexible smoothing with B-splines and penalties. Stat. Sci.

2. **Currie I**, **Durban M**, **Eilers P**. 2004. Smoothing and forecasting mortality rates. Stat. Modelling.

3. **Team R**. 2014. R: A language and environment for statistical computing. R Foundation for Statistical Computing, Vienna, Austria. 2013.

4. **Camarda C**. 2012. MortalitySmooth: An R package for smoothing Poisson counts with P-splines. J. Stat. Softw.

5. **Chaudhuri P**, **Marron J**. 1999. SiZer for exploration of structures in curves. J. Am. Stat.

6. **Jacobs JH**, **Archer BN**, **Baker MG**, **Cowling BJ**, **Heffernan RT**, **Mercer G**, **Uez O**, **Hanshaoworakul W**, **Viboud C**, **Schwartz J**, **Tchetgen Tchetgen E**, **Lipsitch M**. 2012. Searching for Sharp Drops in the Incidence of Pandemic A/H1N1 Influenza by Single Year of Age. PLoS One **7**:e42328.

7. **Sonderegger D**. 2011. SiZer: significant zero crossings. R package version 0.1-4.
